# Magnetic Fields Reduce Apoptosis by Suppressing Phase Separation of Tau-441

**DOI:** 10.34133/research.0146

**Published:** 2023-05-11

**Authors:** Wen-Juan Lin, Wen-Pu Shi, Wan-Yi Ge, Liang-Liang Chen, Wei-Hong Guo, Peng Shang, Da-Chuan Yin

**Affiliations:** Key Laboratory for Space Bioscience and Biotechnology, School of Life Sciences, Northwestern Polytechnical University, 127 Youyixi Road, Xi'an 710072, Shaanxi, PR China.

## Abstract

The biological effects of magnetic fields (MFs) have been a controversial issue. Fortunately, in recent years, there has been increasing evidence that MFs do affect biological systems. However, the physical mechanism remains unclear. Here, we show that MFs (16 T) reduce apoptosis in cell lines by inhibiting liquid–liquid phase separation (LLPS) of Tau-441, suggesting that the MF effect on LLPS may be one of the mechanisms for understanding the “mysterious” magnetobiological effects. The LLPS of Tau-441 occurred in the cytoplasm after induction with arsenite. The phase-separated droplets of Tau-441 recruited hexokinase (HK), resulting in a decrease in the amount of free HK in the cytoplasm. In cells, HK and Bax compete to bind to the voltage-dependent anion channel (VDAC I) on the mitochondrial membrane. A decrease in the number of free HK molecules increased the chance of Bax binding to VDAC I, leading to increased Bax-mediated apoptosis. In the presence of a static MF, LLPS was marked inhibited and HK recruitment was reduced, resulting in an increased probability of HK binding to VDAC I and a decreased probability of Bax binding to VDAC I, thus reducing Bax-mediated apoptosis. Our findings revealed a new physical mechanism for understanding magnetobiological effects from the perspective of LLPS. In addition, these results show the potential applications of physical environments, such as MFs in this study, in the treatment of LLPS-related diseases.

## Introduction

Magnetic fields (MFs) often have a sense of mystery, which is probably because MFs work in a noncontact manner. This becomes even more complicated when MFs encounter biological systems, and quite conflicting views emerge. Some claim that an MF has amazing biological effects, while others dismiss these effects as pseudoscience. Fortunately, in recent years, owing to more rigorous and advanced experimental technologies, more reliable results have been obtained, confirming the reproducibility of MF effects. In addition, several therapeutic effects have been clinically explored and applied. Static MFs, for example, have been found to promote proliferation of osteoblasts, which could be useful in treating osteoporosis [1]. In the study of the effect of MFs on Alzheimer’s disease (AD), it was found that MFs delayed the pathological process [2]. More recently, static MFs have been found useful in the treatment of AD [3]. A technology called repetitive transcranial magnetic stimulation has already been applied in the treatment of neuropsychiatric diseases such as depression, AD, Parkinson’s disease, schizophrenia, and other diseases by providing bioenergy [4,5]. These accumulated experimental results indicate that MFs are potentially applicable for treating biological systems. However, compared with the experimental research, the theoretical studies clearly lag behind. Currently, no satisfactory physical mechanism has been proposed to explain the biological effects of MFs. There is no doubt that studying the mechanism of MF effects on biological systems from the perspective of physics is fundamentally important for better utilization of MFs in practical applications, such as the treatment of diseases.

AD is an age-related, progressive, and destructive neurodegenerative disease with high clinical morbidity [6]. The total cost of treating AD worldwide exceeds trillions of dollars each year [7]. With aging challenges becoming increasingly serious in the world, research on AD treatment technology has become increasingly more important. In the past, researchers conducted a large number of studies on its pathogenesis. To date, it was generally assumed that AD was caused by amyloidosis in the brain, tangling of neuronal fibers, or loss of selective neurons [8,9]. Tubulin-associated unit (Tau) is one of the focused proteins highly correlated with these causes. The aggregation of Tau and phosphorylated Tau leads to the formation of neuronal fiber entanglement [10]. The loss of selective neurons and the reduced efficiency of glucose metabolism are also closely related to the aggregation of Tau proteins [11]. Therefore, aggregated Tau becomes a biomarker of AD [12] and an important target in the study of the pathogenesis of AD.

As a natural and highly soluble unfolded protein, Tau forms a β-sheet structure at physiological concentrations in its microtubule-binding repeat (MTBR) domain. Tau contains an MTBR region, which easily forms β-sheets of fibrous aggregations [13]. In the brains of patients with AD, Tau aggregates to form toxic fibrillary tangles [14]. These pathological processes of Tau aggregation and fibrous structure formation are characteristics of AD. At present, it is known that these processes happen after a process called liquid–liquid phase separation (LLPS). LLPS is not a new phenomenon. It is ubiquitous in physical sciences, such as in phase transitions, solution crystallizations, separations, and extractions. This phenomenon occurring in biological systems is exceptionally important. In 2009, Brangwynne et al. [15] found for the first time that the RNA and protein-rich P granules in the germ cell of *Caenorhabditis elegans* exhibited liquid-like behaviors and proposed that such phase transition behaviors played an important role in structuring the cytoplasm. This discovery revealed a general and fundamental physicochemical mechanism for understanding a variety of cellular physiological and biochemical processes in biological systems, such as gene transcription [16], DNA repair [17], autophagy [18], spindle assembly [19], transcriptional regulation [20], noise reduction in protein concentration in cells [21], RNA metabolism [22], ribosome synthesis [23], DNA damage, and signal transduction [24]. In addition, it has been found that LLPS is also widely involved in many pathological processes, especially in neurodegenerative diseases, such as AD [25], Parkinson’s disease [26], and amyotrophic lateral sclerosis [27]. It has become clear that targeting the LLPS process may offer new opportunities to find solutions to treat LLPS-related diseases.

The LLPS of Tau forms dense liquid droplets containing highly concentrated Tau. These droplets can recruit macromolecules as they form [28]. Immunoprecipitation studies confirmed that Tau also binds to hexokinase (HK) [29,30], which is ubiquitously expressed in the brain and catalyzes the rate-limiting and first irreversible step of glycolysis [31]. HK directly interacts with mitochondrial voltage-dependent anion channel (VDAC I), resulting in a decrease of Bax binding and oligomerization at the mitochondrial contact site. Consequently, Bax-mediated apoptosis signaling is antagonized [32]. After HK dissociation, Bax binds and oligomerizes at the contact site, resulting in decreased mitochondrial membrane potential [33]. A rapid decline in HK activity was observed in the brains of patients with AD [34], suggesting that the number of HK molecules in the cytoplasm was reduced because of the recruitment of HK to the phase-separated droplets.

The phase separation of Tau can be affected by a variety of chemical, biochemical, and physical factors, among which chemical factors are the most widely studied. For example, it was found that at a low Na^+^ concentration (150 mM), the LLPS of Tau was mainly affected by electrostatic interactions [14], while at a high Na^+^ concentration (4 M), it was affected by hydrophobic interactions [35]. The LLPS of Tau is also sensitive to the environment, such as temperature, pH, Na^+^ ion concentration, and salt ion type [36], as well as physical properties such as viscosity and diffusivity [37]. Compared with the chemical environments, the physical environmental factors such as MFs, mechanical vibrations, light, and sound waves have not been comprehensively studied in LLPS research. In other words, the study of the influence of the physical environment on LLPS processes is a research direction that has not been well explored. Because the physical environment, for example, MFs and gravitational fields, affects solution properties, such as viscosity [38], wettability [39], and diffusivity, as well as other parameters, we expect that the physical environment will have an impact on LLPS, thus further affecting the biological processes associated with LLPS. This is a very important inference because it provides a plausible physical mechanism for understanding the mysterious effects of MFs on biological systems and, furthermore, indicates that physical environments can be used to treat LLPS-related diseases.

In this study, we constructed 293T-TAU441 and SK-N-SH-TAU441 cell lines in which the LLPS of Tau-441 occurred after induction with arsenite. An MF (16 T) inhibited the LLPS of Tau-441, which reduced the recruitment of HK by the phase-separated droplets, resulting in more HK molecules remaining in the cytoplasm than without MF exposure. The greater the number of HK molecules in the cytoplasm, the greater was the chance of HK binding to VDAC I, which thereby reduced Bax-mediated apoptosis. Our study provides a novel physical mechanism for understanding how an MF affects biological systems. In addition, this work also showed that physical environments such as MFs can be explored for the treatment of LLPS-related diseases.

## Results

### Tau-441 phase separation interacts with HK

**Fig. 1.**
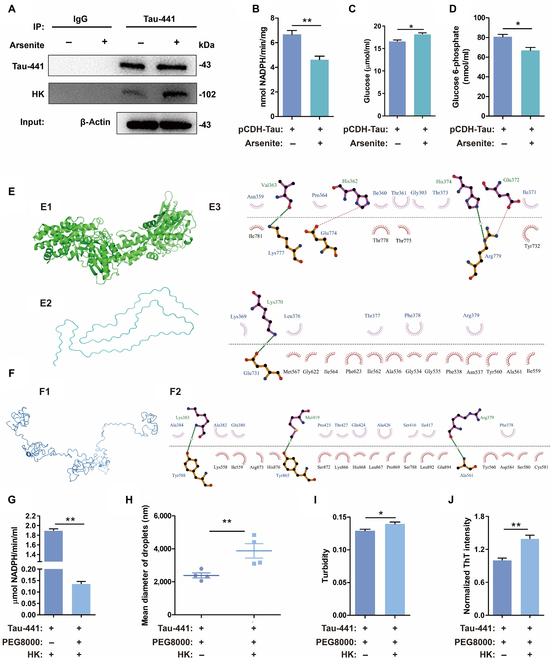
LLPS of Tau-441 enhanced its binding to HK. (A) Western blotting of Tau-441, hexokinase (HK), and β-actin expression in the lysate and Co-immunoprecipitation (Co-IP) of 293T-TAU441 cells. (B) HK activity indicated by the levels of NADPH (reduced form of nicotinamide adenine dinucleotide) in cells with and without induction by 0.5 mM arsenite. (C and D) Amounts of glucose and glucose 6-phosphate in the cells with and without arsenite induction. (E) Putative binding sites between HK and the MTBR domain of Tau-441. (E1) Crystal structure of human HK binding with glucose and ADP in active sites from the PDB database [40]. (E2) Fragment structure of 4R tauopathy type 1b Tau filament from the PDB database [41]. (E3) The putative binding sites between Tau-441 and HK. The dotted green lines indicate hydrogen bonds, and the arc with spokes radiating toward the ligand atoms indicates hydrophobic interactions. (F) Putative binding sites between HK and homologous modeling of native Tau-441. (F1) Structure of homologous modeling of native Tau-441 given by I-TASSER online protein model software. (F2) Putative binding sites between Tau-441 and HK. The dotted green lines indicate hydrogen bonds, and the arc with spokes radiating toward the ligand atoms indicates hydrophobic interactions. (G) HK activity in the mixed solution of HK and Tau-441 with and without LLPS. LLPS of Tau-441 was induced by adding 10% PEG8000 at 310.15 K for 30 min. (H) Size of phase-separated droplets in solutions of Tau-441 with and without adding HK. (I and J) Turbidity and normalized thioflavin T (ThT) intensity of Tau-441 solution with and without adding HK. Data were shown as means ± SEM. *n* = 3 to 7 per group. *P < 0.05 and **P < 0.01. IgG, immunoglobulin G.

The phase separation of Tau-441 was induced by mixing arsenite with 293T-TAU441 cells for 0.5 h. The phase separation induced by arsenite did not increase the expression of Tau. However, the number of HK molecules binding to Tau-441 increased, as indicated by the Western blotting result (Fig. 1A). Because the main function of HK is to catalyze glucose in the glycolysis pathway, we detected changes in HK activity in cells by monitoring the amount of reduced form of nicotinamide adenine dinucleotide phosphate (Fig. 1B). The amount of glucose, which is the substrate for HK, increased (Fig. 1C), while the product glucose 6-phosphate decreased (Fig. 1D). These results showed that arsenite reduced the activity of HK in 293T-TAU441 cells.

A Co-IP (co-immunoprecipitation) experiment (Fig. 1A) showed that intracellular Tau-441 bound to HK, and phase separation increased the binding of HK to Tau-441. We further used the molecular docking software ZDOCK [42] to simulate the binding mode of Tau-441 with HK. ZDOCK scores every possible pose in which 2 proteins bind on the basis of potential energy, spatial complementarity, and an electric field force. According to the scores, we chose the top 10 poses for analysis. LigPlot software was used to determine the formed hydrogen bonds and hydrophobic interactions [43]. The MTBR region is considered to be an important region for Tau-441 aggregation, and therefore, we selected the structure of the MTBR region (Fig. 1E2) from the Protein Data Bank (PDB) database to dock with HK (Fig. 1E1). The docking result showed that HK interacted with Tau-441 mainly through hydrophobic interactions and intermolecular hydrogen bonds. Figure 1E3 shows one of the combination forms. It can be seen that Val363, His362, His374, Glu372, and Lys370 of the MTBR domain of Tau-441 formed hydrogen bonds with Lys777, Glu774, Arg779, Arg779, and Glu731 of HK, respectively. Figure S1 shows the other 9 results that demonstrated that hydrogen bonds and hydrophobic interactions occurred between the 2 proteins. In addition to the structurally altered Tau-441 with the MTBR domain, the native Tau-441 also existed in solution. Therefore, we also analyzed the binding of HK to native Tau-441. I-TASSER [44], an online protein modeling software, was used to construct the homology model of Tau-441. The confidence score (*C* score) structure was selected for subsequent molecular docking analysis. Figure 1F1 shows the simulated molecular structure of Tau-441. The results showed that HK also bound to native Tau-441 (Fig. 1F2 and Fig. S2). The stability of the protein structure was positively correlated with the value of the solvation free energy. We used the online software PDBePISA [45] to calculate the solvation free energy gains between HK and native Tau-441. The results are presented in Table S1.

Arsenite induces LLPS of intracellular Tau-441, which may cause changes in intracellular metabolism. To eliminate the interference of other intracellular factors in the cell, we incubated Tau-441 directly with HK in solution for an LLPS experiment. It was found that the activity of HK significantly decreased when incubated with Tau-441 in a solution with 10% PEG8000 (polyethylene glycol, molecular weight 8000) than when incubated with Tau-441 in a solution without 10% PEG8000 (Fig. 1G). These results revealed that the phase separation of Tau-441 decreased the overall activity of HK in the solution by increasing its binding to HK.

According to the structure of HK in the PDB database (1DGK), we can see the active sites of glucose, adenosine diphosphate (ADP), and a phosphate ion combined with HK, as shown by LigPlot (Fig. S3). It was shown that Thr680 and Ala536 of HK were not only the sites binding to ADP but also the sites binding to Tau-441 via hydrogen bonds. This may be the reason for the decrease of HK activity caused by Tau-441 binding.

Through the above experiments, we confirmed the recruitment of HK by the LLPS of Tau-441. The LLPS of Tau-441 may also be affected by certain conditions, including inhibitors, facilitators, and exogenous macromolecules such as PEG during the formation of phase separation droplets. Therefore, we analyzed whether the recruited HK promoted or suppressed Tau-441 LLPS. To find the answer, we studied the turbidity, particle size, and β-sheet content, which are effective indicators for characterizing protein LLPS. We obtained the changes of these indicators after HK was added to the solution. Under the influence of PEG, both particle size and turbidity increased with an increasing amount of HK (Fig. 1H and I). The size of the particles in the solution increased significantly from 2.39 ± 0.33 μm to 3.88 ± 0.88 μm with HK (Fig. 1H). Furthermore, the addition of HK increased the turbidity of the solution from 0.13 ± 0.0033 to 0.14 ± 0.0039 (Fig. 1I). The fluorescence intensity of thioflavin T (ThT) in the Tau-441 solution also increased, indicating an increase in β-sheet content (Fig. 1J). The addition of HK increased the fluorescence intensity of the Tau-441 solution compared with that of the solution without HK. These results indicated that the addition of HK augmented the LLPS of Tau-441. This may be because HK stabilized the β-sheet structure of Tau by hydrogen bonding with the MTBR region of Tau-441.

### The MF-inhibited phase separation of Tau-441

**Fig. 2.**
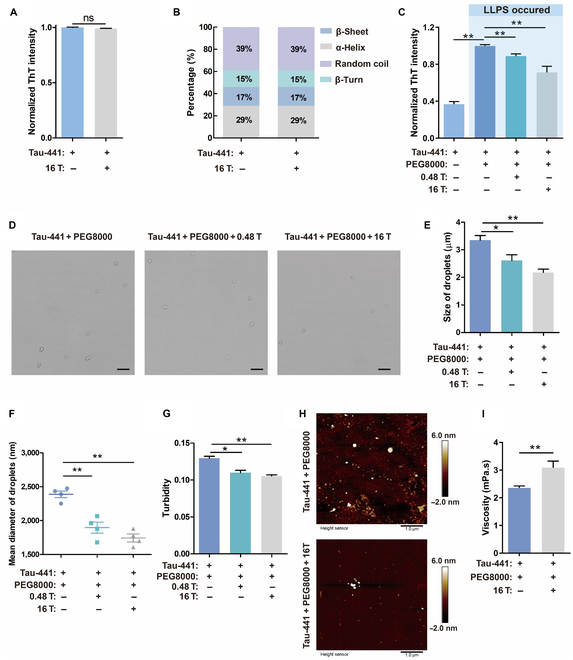
MFs inhibited LLPS of Tau-441. (A) An MF (16 T) effect on the formation of β-sheet by ThT fluorescence intensity study. Tau-441 was incubated in a buffer solution (150 mM NaCl and 10 mM Hepes (pH 7.4)) without LLPS occurred. Results were normalized as the fluorescence intensity/fluorescence intensity of protein without an MF (16 T). (B) The change of the secondary structure content of protein determined by circular dichroism. Tau-441 was incubated in buffer solution (150 mM NaCl and 10 mM Hepes (pH 7.4)) without LLPS induction. (C) Inhibition effect of static MFs on LLPS verified by ThT fluorescence intensity comparison. (D) Tau-441 formed liquid droplets under different conditions. Scale bars, 10 μm. (E) The droplet sizes in the field of view obtained by ImageJ analysis on the microscopic pictures in (D). (F) The mean diameter of Tau-441 droplets. *n* = 4 per group. (G) MF inhibition effect on LLPS verified by turbidity. (H) AFM images of the Tau-441 clusters with and without an MF (16 T) treatment. Scale bars, 1 μm. (I) An MF (16 T) effect on viscosity. Data were shown as means ± SEM. *n* = 3 to 6 per group. *P < 0.05 and **P < 0.01. ns, not significant.

The schematic illustration of magnet systems and MF distribution are shown in Fig. S4. We examined the ThT intensity of the native Tau-441 dissolved in the buffer solution when no LLPS occurred. It was found that there was no significant difference in the ThT intensity of the samples treated with and without an MF (16 T, superconducting magnet) (Fig. 2A), confirming that the MF (16 T) did not affect the β-sheet content of native Tau-441. We further used circular dichroism (CD) spectroscopy to analyze the effect of a 16-T MF on the secondary structure of native Tau-441. The results (Fig. 2A and B) showed that the MF (16 T) did not change the secondary structures of native Tau-441.

Although the secondary structure of native Tau-441 did not change in the MF (16 T) without the occurrence of LLPS, as demonstrated above, this was not the case when LLPS occurred. We found that LLPS caused an apparent change in secondary structure (Fig. 2C). The β-sheet content in the Tau-441 sample increased significantly after LLPS as compared with the native Tau-441 without LLPS. Furthermore, both high (16 T, superconducting magnet) and low (0.48 T, permanent magnet) MFs attenuated the increase in β-sheet content (Fig. 2C). We hence examined whether an MF (16 T) could reduce the effect of LLPS on the secondary structures of Tau-441. The results (Table 1) showed that, in the presence of an MF (16 T), the β-sheet content (27.38% ± 0.79%) was less than that in the absence of an MF (31.03% ± 0.20%), and the α-helix content (13.21% ± 0.35%) was greater than that in the absence of an MF (8.18% ± 0.46%). These results show that the secondary structure of Tau-441 changed more significantly in the absence of an MF than in the presence of an MF (16 T), revealing the stabilizing effect of an MF on the secondary structure of Tau-441.

**Table 1.** MF treatment (16 T) mitigated changes in the secondary structure of Tau-441 exacerbated by LLPS. The samples were Tau-441 solutions after LLPS occurred. In both cases (with and without MF treatment), the secondary structure of Tau-441 changed because of LLPS. The data presented in this table show that the MF slowed down the changes in the secondary structure, providing evidence of the stabilizing effect of the MF on Tau-441 structure.

Secondary structures	No MF (%)	MF (%)
α-Helix	8.18 ± 0.46	13.21 ± 0.35**
β-Sheet	31.03 ± 0.20	27.38 ± 0.79**
β-Turn	16.92 ± 0.70	17.71 ± 0.35
Random coil	43.87 ± 0.58	41.66 ± 0.89**

Data were shown as the mean ± SEM. *n* = 3 per group. **P < 0.01.

LLPS is a continuous process. To reduce the experimental error caused by long-term microscope monitoring after the droplets were removed from MFs, glutaraldehyde was used to cross-link the droplets to ensure that the droplet shapes remained unchanged during microscope. After fixing with glutaraldehyde, the phase-separated droplets of Tau-441 with PEG8000 were observed under a microscope. It was found that the number of droplets formed in the presence of both high (16 T, superconducting magnet) and low (0.48 T, permanent magnet) MFs was less than the number of droplets formed in the absence of MFs (Fig. 2D). The statistical analysis of droplets in the photos showed that there were 11 droplets formed in the 16-T MF, 15 droplets formed in the 0.48-T MF, and 23 droplets formed without an MF. We used a microscope and ImageJ statistics to study the size of droplets in the field of view and found that the size of the droplets obtained with the MFs (0.48 and 16 T) was significantly smaller than that of the droplets obtained without the MF (Fig. 2E). To further confirm this observation, we used dynamic light scattering (DLS) to obtain the average droplet size in the entire solution system. The mean diameter of Tau-441 droplets obtained without the MF was 2.39 ± 0.08 μm, that of Tau-441 droplets obtained with the 0.48-T MF was 1.90 ± 0.14 μm, and that of Tau-441 droplets obtained with the 16-T MF was 1.74 ± 0.10 μm (Fig. 2F). To eliminate unrelated interferential factors, various methods were used for further verifying the influence of MFs on Tau-441 phase separation. We observed a rapid decrease in sample turbidity with the MF (0.48 and 16 T) compared to that without the MF, which strongly indicated that the MF was involved in the inhibition of LLPS (Fig. 2G).

We diluted (1:10,000) the phase-separated solutions and pipetted a drop onto a silicon wafer and then removed the excess solution with a filter paper. Then, we used an atomic force microscope (AFM) to examine the particles remaining on the silicon surface. Interestingly, it was found that the number of particles was much less in the sample with the MF than in the sample without the MF (38 versus 211), indicating that there was already partial liquid–solid phase transition occurring in the phase-separated droplets, and the transition was significantly faster or earlier in the absence of the MF than in the presence of the MF (Fig. 2H). This result was additional evidence that the LLPS of Tau-441 was inhibited by an MF.

Solution viscosity is known to be inversely proportional to the phase separation process. Increasing viscosity should inhibit LLPS. We measured the viscosity of the Tau-441 solution and confirmed that the MF (16 T) significantly increased the viscosity of the solution (Fig. 2I).

### The MF (16 T) inhibited the phase separation of a solution incubated with Tau-441, reduced the binding of HK to Tau-441, and increased the content of free HK and the HK activity

**Fig. 3.**
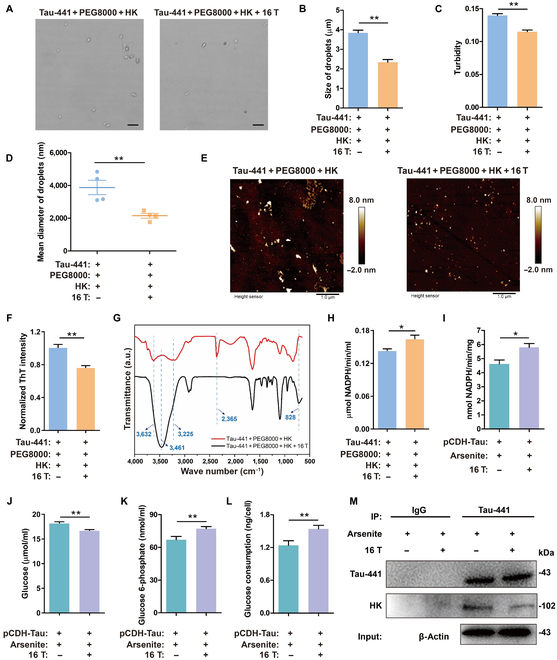
An MF (16 T) treatment disrupted phase separation and increased HK activity. (A) An MF (16 T) effect on droplet formation of Tau-441 after incubation with HK. Scale bars, 10 μm. (B) The droplet sizes in the field of view obtained by ImageJ analysis on the microscopic pictures in (A). (C) An MF (16 T) inhibition effect on LLPS verified by turbidity. (D) The mean diameter of Tau-441 droplets. *n* = 4 per group. (E) AFM images of the Tau-441 clusters with and without an MF (16 T) treatment. Scale bars, 1 μm. (F) ThT fluorescence intensity of the phase-separated solution with and without an MF (16 T) treatment. (G) Fourier transform infrared spectra of mixed solution of HK and Tau-441 after LLPS induction treatment using PEG8000 at 310.15 K. (H) An MF (16 T) increased HK activity in Tau-441 phase separation solution. (I) An MF (16 T) increased HK activity in cells with Tau-441 phase separation. (J to L) Amounts of glucose, glucose 6-phosphate, and glucose consumption in cells with and without an MF (16 T). (M) Western blotting of Tau-441, HK, and β-actin expression in lysate and Co-IP of 293T-TAU441 cells. Data were shown as means ± SEM. *n* = 3 to 7 per group. *P < 0.05 and **P < 0.01. a.u., arbitrary units.

To further understand the role of the MF (16 T) in the formation of Tau-441 and HK binding, the droplet size, the turbidity, and the size of clusters in the solution were studied with and without an MF (16 T). The results revealed that all 3 parameters were significantly reduced under an MF (16 T) (Fig. 3A to D). Novel differential interference contrast (DIC) microscope images showed that the droplets formed in the presence of an MF (16 T) were much less than those formed in the absence of an MF (Fig. 3A). Using the software ImageJ, we found that the size difference was statistically significantly (*P* < 0.01) (Fig. 3B). The statistical analysis of droplets in the photos showed that there were 13 droplets formed with the MF (16 T) and more than 31 droplets formed without the MF. Moreover, the turbidity of the solution with Tau-441 and HK was lower in the MF (16 T) (0.12 ± 0.0036) than that without the MF (0.14 ± 0.0039) (Fig. 3C). Through DLS, we further confirmed that the average size of the droplets formed with the MF (16 T) was smaller (2.15 ± 0.88 μm) than that of the droplets formed without the MF (3.88 ± 0.88 μm) (Fig. 3D). The number of clusters after dilution of the phase-separated solution was significantly lower (*n* = 103) with the MF (16 T) than without the MF (*n* = 166) (Fig. 3E). All the above results verified that the MF (16 T) suppressed the LLPS of Tau-441 with added HK.

To further understand the MF (16 T) effect on the binding between Tau-441 and HK, the secondary structure in the solution was studied with and without an MF (16 T). The fluorescence intensity of ThT was used to represent β-sheet content. It can be seen that the fluorescence intensity with the MF (16 T) was less than that without the MF, demonstrating that the β-sheet content with the MF (16 T) was less than that without the MF (Fig. 3F). Furthermore, the α-helix content with the MF (16 T) was significantly greater than that without the MF (Table 2). These results again confirmed that the secondary structure of Tau-441 with HK added after LLPS changed more slowly under an MF (16 T) than without an MF, indicating the protective effect of an MF on the secondary structure during LLPS.

**Table 2.** MF (16 T) treatment mitigated changes in the secondary structure of Tau-441 when HK was added to a Tau-441 solution following LLPS. HK was added to the Tau-441 solutions after LLPS occurred. In both cases (with and without MF), the secondary structure of Tau-441 changed because of LLPS. The data presented in this table show that the MF slowed down the changes in the secondary structure, providing additional evidence of the stabilizing effect of the MF on Tau-441 structure.

Secondary structures	No MF (%)	MF (%)
α-Helix	5.62 ± 0.67	8.71 ± 0.34**
β-Sheet	34.10 ± 0.42	30.45 ± 0.02**
β-Turn	16.61 ± 0.67	16.89 ± 0.79
Random coil	43.68 ± 0.62	43.98 ± 0.67

Data were shown as the mean ± SEM. *n* = 3 per group. **P < 0.01.

To further study the binding between the 2 molecules under the influence of an MF (16 T), Fourier transform infrared spectroscopy (FTIR) was used. Figure 3G shows the effect of the MF (16 T) on the FTIR spectra of the mixed solution of HK, Tau-441, and PEG8000. The change of the peak in the FTIR spectra is related to the vibration of molecular groups [46]. The range from 2,500 to 3,700 cm^−1^ in the FTIR spectra was the hydrogen stretching region, in which the vibration frequencies of C–H, N–H, and O–H appeared [47]. The peak at 2,365 cm^−1^ is the stretching region of C≡C. The range from 400 to 1,000 cm^−1^ is the stretching region of aromatic bonds [48]. From the FTIR spectra comparison, it can be seen that the main changes under MF (Fig. 3G) were the disappearance of peaks at 3,632, 3,225, and 2,365 cm^−1^ and the appearance of peaks at 3,461 and 828 cm^−1^. As can be seen from Fig. 2B and Table 1, the MF did not change the secondary structure of native Tau-441 but played a role in slowing the changes in secondary structure. The breaking of hydrogen bonds and formation of new bonds were clear signals that regulated the LLPS process [49]. Because the peak at 3,461 cm^−1^ was related to C–H, that at 2,365 cm^−1^ was related to C≡C, and that at 828 cm^−1^ was related to aromatic bonds, the FTIR comparison results indicated that the C–H bond and aromatic bonds were broken, and the C≡C bond formed after LLPS under the action of the crowding agent (PEG8000). Under MF treatment, such changes in the bonding were suppressed (Fig. 3G) compared with the Tau-441 solution without LLPS (refer to the FTIR spectrum of the Tau-441 solution; Fig. S5). The peaks at 3,632 and 3,225 cm^−1^ represented the intermolecular hydrogen bonds [50]. Our docking studies in Fig. 1E and F showed that HK bound to Tau-441 through hydrogen bonds, thereby exacerbating the LLPS of Tau-441 (Fig. 1H to J); hence, these peaks were observed in the FTIR spectrum of samples without MF treatment. In the samples with MF treatment, these peaks disappeared, which was similar to the case of the Tau-441 spectrum without LLPS (see Fig. S5), indicating that the formation of intermolecular hydrogen bonds was suppressed by the MF.

The decreased HK recruitment by LLPS droplets resulted in an increase of free HK in the solution. Compared with the case without an MF, the activity of HK in the solution was significantly enhanced with an MF (16 T) (Fig. 3H). Meanwhile, the activity of intracellular HK also significantly increased with the MF (16 T) (Fig. 3I). The amount of glucose decreased (Fig. 3J) and the amount of glucose 6-phosphate increased (Fig. 3K). The enhancement of HK activity also improved the utilization rate of glucose by cells (Fig. 3L). These results suggested that an MF (16 T) improved glucose metabolism. Although the MF (16 T) showed no effect on the expression of Tau-441 in the cells after arsenite-induced phase separation, the amount of HK bound to Tau-441 in the MF (16 T) was significantly reduced (Fig. 3M).

### HK can competitively bind to VDAC I and inhibit cell apoptosis

**Fig. 4.**
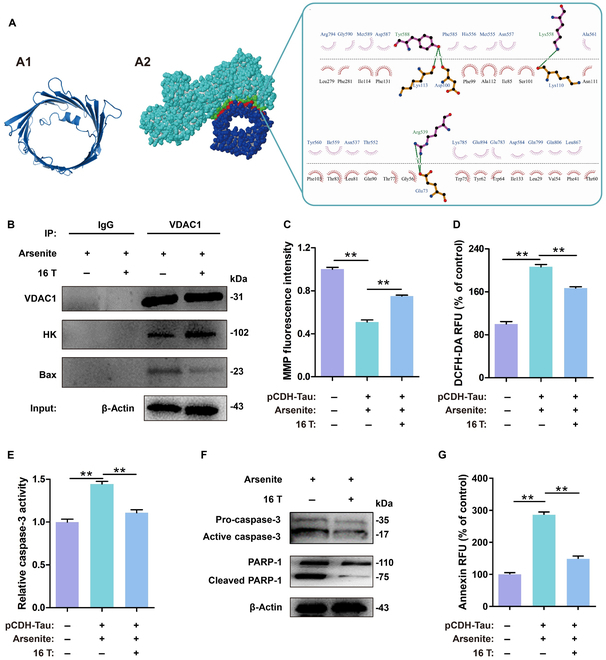
Increase in the number of free HK molecules due to an MF (16 T) decreased the chance of Bax binding to VDAC I, leading to decreased Bax-mediated apoptosis. (A) Putative binding sites between HK and VDAC I. (A1) Structure of VDAC I from the PDB database (6G73). (A2) Putative binding sites between VDAC I and HK. Green spheres indicate the residue of HK at the interaction site between HK and VDAC I. Red spheres indicate the residue of VDAC I at the HK–VDAC I interaction site. The dotted green lines indicate hydrogen bonds, and the arc with spokes radiating toward the ligand atoms indicates hydrophobic interactions. (B) Western blotting of VDAC I, HK, Bax, and β-actin expression in lysate and Co-IP of 293T-TAU441 cells. (C) Mitochondrial membrane potential (MMP) measured using a fluorescence spectrophotometer with and without an MF (16 T). (D) The DCFH-DA fluorescence intensity representing ROS generation in 293T-TAU441 cells with and without an MF (16 T). (E) Caspase-3 activity with and without an MF (16 T) measured by a fluorescence of Ac-DEVD-pNA. (F) Western blotting assay showed levels of active caspase-3 and cleaved PARP-1. (G) Cell apoptosis with and without an MF (16 T) detected by Annexin V-PE. Data were shown as means ± SEM. *n* = 3 per group. **P < 0.01. RFU, relative fluorescence units.

Free HK also binds to native Tau-441 in solution. Therefore, we used molecular docking to simulate the docking between HK and VDAC I. The results gave possible poses, among which the top 10 were examined. We found that several binding sites between HK and VDAC I have been reported in the literature [51]. Figure 4A shows 1 pose, and the other 9 poses are shown in Fig. S6. The free energy gains corresponding to these poses are presented in Table S2. The results showed that most of the solvation free energy gains upon HK binding to VDAC I were less than those upon HK binding to native Tau-441, indicating that HK was more likely to bind to VDAC I. To test whether Tau-441 under the influence of the MF (16 T) was involved in this apoptotic process, we performed Co-IP experiments. The results showed that, under an MF (16 T), the amount of intracellular VDAC I that bound to HK increased, while the amount of intracellular VDAC I that bound to Bax decreased (Fig. 4B).

We analyzed the apoptosis of 293T-TAU441 cells treated with an MF (16 T) for 6 and 24 h (Fig. S7 and Fig. 4). In the early stage of apoptosis, Bax opens mitochondrial VDAC I, changes the membrane potential, and releases various proapoptotic factors. Therefore, we used JC-1 to detect membrane potential changes and quantified them by detecting green and red fluorescence intensities. The results showed that an MF (16 T) increased the fluorescence of the mitochondrial membrane (Fig. 4C), indicating that the damage was significantly reduced. Arsenite promotes the production of mitochondrial reactive oxygen species (ROS) [52]. Excessive ROS can induce the opening of mitochondrial membrane pores, thus releasing cytochrome C and apoptosis-inducing factors and enabling caspase-9 to activate caspase-3/6/7. Regulation of ROS has been used to treat various diseases [53]. A decrease in the intracellular ROS content (Fig. 4D) was observed with the MF (16 T) as compared to that without the MF. The activity of intracellular caspase-3 in 293T-TAU441 cells was significantly reduced under the MF (16 T) for 24 h (Fig. 4E), showing that the MF (16 T) reduced apoptosis. Consistent with the results of an enzyme activity assay, the MF (16 T) reduced the expression of the active form of caspase-3 and inhibited the cleavage of the substrate protein poly ADP-ribose polymerase (PARP-1) degraded by activated caspase-3 (Fig. 4F). The MF (16 T) reduced the number of early apoptotic cells, as shown by an Annexin V-phycoerythrin (PE) analysis (Fig. 4G).

To further confirm the observations, we constructed another cell line, SK-N-SH-TAU441, and experimentally examined the effect of the MF (16 T) on cell apoptosis during LLPS. After overexpression of Tau-441, LLPS was induced in the cells. The effect of the MF (16 T) on apoptosis was also analyzed at 6 and 24 h. This experiment resulted in the same conclusion, that is, the MF (16 T) inhibited apoptosis of SK-N-SH-TAU441 cells at 6 h (Fig. S8) and 24 h (Fig. S9).

## Discussion

### How does an MF affect the LLPS of Tau-441?

Fundamentally, there are several possible mechanisms for understanding the effect of an MF on LLPS.

#### MF effects from the perspective of energy: MFs show discernable effects on a phase-separated droplet when its size is sufficiently large

Every substance has a magnetic free energy *E*_mag_ under the action of an MF, and when the components involved in the phase transition have the same magnetism, the change in *E*_mag_ is written [54]:

$$E_{\mathrm{mag}}=\frac{\Delta \chi VB^{2}}{2\mu_{0}}$$ (1)

assuming that the energy of a phase-separated droplet under an applied MF is *E*_mag_.

where *V* is the volume of the droplet, *B* is the applied MF, Δ*χ* is the difference in the magnetic susceptibility between the phase-separated droplet and the surrounding bulk solution, and *μ*_0_ is the magnetic permeability in vacuum.

The phase-separated droplet is subjected to both thermal energy and MF effects. To ensure that the MF exerts a discernable effect on the droplet, the energy of the droplet in the MF must overcome the thermal energy *k*_B_*T*, i.e., *E*_mag_ > *k*_B_*T*:

$$\frac{\Delta \chi VB^{2}}{2\mu_{0}}>k_{B}T$$ (2)

where *k*_B_ is the Boltzmann constant, and *T* is the temperature.

Replacing the droplet volume *V* with the droplet size *r*, we have:

$$\frac{\Delta \chi VB^{2}}{2\mu_{0}}=\frac{\Delta \chi \left(\frac{4}{3}{\pi r}^{3}\right)B^{2}}{2\mu_{0}}>k_{B}T$$ (3)

From Eq. 3, we can derive the following formula:

r>3μ0kBT2πΔχB213=rc (4)

Equation 4 shows that, when the size of the droplet exceeds a critical value *r_c_*, discernable effects due to the MF will occur. If the droplet size is too small (smaller than *r_c_*), then the MF effect may not be observable.

We can estimate the critical size of the droplet from Eq. 4. The magnetic susceptibility difference Δ*χ* between the droplet and the surrounding environment can be measured experimentally (Δ*χ* = 1.2 × 10^−6^; see “Supporting method 2” in the Supplementary Materials). If we choose the temperature *T* = 310.15 K (37 °C) and use the constants *k*_B_ = 1.380649 × 10^−23^ J/K and *μ*_0_ = 4 × 10^−7^ N/A^2^, then in the case when *B* = 16 T, *r_c_* = 20.3 nm and when *B* = 0.48 T, *r_c_* = 210.2 nm, it can be seen that the stronger the MF, the smaller the critical size of the droplets.

The above theoretical derivation shows that the MF can interfere with the LLPS process when the phase-separated droplet size is sufficiently large. If the droplet size is too small, then the thermal energy will disrupt the MF effect so that no discernable difference from the MF is observed. This effect can explain why the experimental conditions were only slightly different but the results are quite different, thus triggering the controversy over the MF effect on biological systems.

#### MF effect from the perspective of forces: Forces due to MF can slow the LLPS process

1. **Lorentz force**

MFs are known to act on charged particles. When its direction of motion is not parallel to the direction of the MF, the charged particles are subjected to a Lorentz force. Biological macromolecules (such as Tau-441, HK, and Bax) and biomolecular condensates (phase-separated droplets) can be considered as charged species. Under the influence of a Lorentz force, the direction of motion of the charged particles will deviate. In the case in which a charged particle enters perpendicular to a uniform MF, the radius of the deviation path, *r*, can be determined on the basis of the following equation:

$$r=\frac{\mathrm{mv}}{\mathrm{qB}}$$ (5)

where *m* is the mass of the particle, *v* is the particle velocity, *q* is the electric charge, and *B* is the MF. We can roughly estimate the radius of the deviation path and thus predict what may happen when the MF affects the motion of the macromolecules. In the case of Tau-441, the molecular weight is 43 kDa, the net charge is about +8 [55], and the velocity *v* = 7.7 m/s, according to the prediction equation for the instantaneous velocity of a Brownian particle [56] at temperature *T* = 310.15 K (37 °C).

$$v=\sqrt{\frac{k_{B}T}{m}}$$ (6)

Here, when in the MF, *B* = 16 T and the radius *r* = 26.8 μm. This radius is comparable to the size of a cell, indicating that the MF can influence the motion of the charged macromolecules, thus affecting their physical properties. Because of deviation of the macromolecules, their diffusion will be slowed down, and thus, the diffusion coefficient decreases. This phenomenon has been observed experimentally [57]. On the basis of published experimental data [57], we can estimate the diffusion coefficient of a model protein, lysozyme. The estimated diffusion coefficient of lysozyme in an MF of 6 T is about *D_m_* = 1.60 × 10^−10^ m^2^/s, and in the absence of an MF, *D* = 1.84 × 10^−10^ m^2^/s. The quantitative results confirmed that the diffusion coefficient of macromolecules was reduced by the MF. According to the Stokes–Einstein equation, viscosity increases in the presence of an MF. This assumption was also observed in in situ experiments [58]. It is also known that the solution viscosity is inversely proportional to the phase separation process [37]. Increasing viscosity suppresses the LLPS. Hence, the entire LLPS process of Tau-441 is affected by the Lorentz force.

We also measured the viscosity of a Tau-441 solution after treatment with and without an MF (16 T). It was found that the viscosity of the solution was higher after treatment with an MF (16 T) than without an MF (Fig. 2I), indicating that, in the absence of an MF, LLPS was enhanced, and hence, the bulk solution was diluted, resulting in a lower viscosity. This result is consistent with the reported phenomena observed for cytoplasmic viscosity in the cells of patients with AD. The researchers found a decrease in cytoplasmic viscosity in the hippocampus of patients with AD and AD mouse models [59] and also found a positive correlation between decreased cytoplasmic viscosity and Aβ-amyloid aggregation.

Because the cell is very small, the mass transfer inside is basically diffusive [60]. When the diffusion coefficient decreases, the molecular transport process slows down, which, in turn, slows down the phase separation process that requires molecular diffusion to aggregate biological macromolecules into the concentrated liquid phases (i.e., the phase-separated droplets). Therefore, MFs have a physical basis for slowing down the LLPS of biological macromolecules.

In addition to their effects on diffusivity and viscosity, MFs also affect the motion of phase-separated droplets. The phase-separated droplets can be also considered as charged particles, which move in an MF and are also subjected to a Lorentz force. The Lorentz force slows the movement of these droplets, thereby reducing the chance of collisions between the droplets. Because coalescence resulting from collisions is a very important means by which the droplets grow in size, fewer collisions mean fewer chances for the growth. Therefore, we observed in the experiment that the size of the phase-separated droplets observed in the MF was smaller, more uniform, and distributed in a narrower range as compared with that of the droplets not exposed to the MF (16 T) (Figs. 2F and 3D).

2. **Magnetic force**

In addition to the Lorentz force, all substances are subjected to a magnetic force in a gradient MF. The magnetic force can be obtained using the following equation:

$$\mathrm{Fz}=V\frac{\chi }{\mu_{0}}B\frac{\partial B}{\partial X}$$ (7)

where *F_z_* is the magnetic force in the direction of *z*, *V* is the volume of the object, *χ* is the volume magnetic susceptibility, *μ*_0_ is the magnetic permeability in a vacuum (which is a constant), and *B* is the MF. Note that this force is only generated when there is a gradient in the MF. In reality, MF gradients are ubiquitous. Therefore, the magnetic force should be considered. As discussed above, the magnetic force has an effect if the droplet size is sufficiently large. In an MF, the droplets cannot move as freely as they do in the absence of an MF when the droplet size is sufficiently large; so, the chance of droplets moving and colliding with each other is reduced, resulting in smaller and more uniform-sized droplets of the concentrated liquid phase. This is another explanation to the more uniform and smaller-sized phase-separated droplets observed in the MF (Figs. 2F and 3D).

3. **Magnetic torque**

An object with magnetization *M* will be affected by a torque *T* in an MF *B*:

T=−VMBsinθ (8)

where *V* is the volume of the object and *θ* is the angle between *M* and *B*.

For a single molecule, *M* and *V* are very small, and hence, the torque will be extremely small, so that the single molecule does not orient in the MF. However, when molecules are arranged in a particular way to form a structure, such as amyloid fibers, a sufficiently large MF and a sufficiently regular arrangement of fibers may cause the object to orient in the MF [61,62]. However, in this study, we focused on the effects of the MF (16 T) on phase separation, which is mainly concerned with liquids. Therefore, we tend to believe that an MF does not affect phase separation through torque.

### Indirect evidence for the inhibitory effect of MF on LLPS

The mechanism of MF inhibiting phase separation was discussed from the theoretical point of view and verified by direct experimental results. In addition to the direct results, there is also indirect evidence found in our study for the effect of an MF (16 T) on phase separation.

#### Evidence that phase separation exacerbates secondary structure transitions

In our experimental studies, we found that phase separation exacerbated the transition of secondary structures (Fig. 2C). A possible mechanism is that, under higher concentration conditions, Tau-441 proteins are more likely to interact with each other, which reduces the energy barrier for structure formation and then promotes the formation of a β-sheet structure, so that more obvious secondary structural changes are observed. In our experimental study, we found that the secondary structure changes were slowed down in the presence of an MF (16 T) compared with those in the absence of an MF (Fig. 2C and Table 1). This result verified that an MF (16 T) inhibited the phase separation and, thus, inhibited the secondary structure transition on Tau-441 structure.

It is hard to influence the protein structure (including higher-order protein structure) by artificial static MFs (up to 45 T), because the magnetic energy on the protein molecules exerted by the MFs is far smaller than thermal energy [54]. This is the theoretical basis for understanding the structural stability of protein molecules in static MFs. Another evidence is that there is no significant impact of MFs on the molecular structure of proteins as can be concluded from the studies of growing high-quality protein crystals using high MFs [63,64]. Hence, the difference in the secondary structure of Tau-441 with and without MFs shall be caused by other reasons.

In our research, what we have found was that, when the structure of Tau-441 was changing during LLPS, applying MFs slowed down that structural change. As stated above, MFs could not directly change the structure of a single protein molecule; such slowing down effect shall be caused by magnetic inhibition of LLPS, as shown in Fig. 2C. On the basis of our results, we suggest that the LLPS be avoided in sample preparation for structural determination using NMR.

One more thing needed to point out was that, in our study, we found that MFs help stabilize the Tau-441 structure. Because there are always protein structure changes that occur in the biological systems, this phenomenon may also provide an explanation for some of the biological effects caused by MFs. However further research is necessary to clarify the mechanism.

#### Evidence in liquid–solid phase transition

Further liquid–solid phase transition may occur after LLPS. The insoluble material obtained after dilution of the phase separation solution can be considered as the solid component that completes the liquid–solid transition. The larger the solid fragments, the earlier the phase separation occurred. The solid fragments obtained under MF (16 T) conditions were small (without and with HK; Figs. 2H and 3E, respectively). The results indicate that the MF (16 T) inhibited the phase separation, thus slowing down the subsequent liquid–solid phase transition, so that the solid fragments become smaller.

### Theoretical basis and future perspectives of using MF as a potential tool to intervene with LLPS-related diseases

AD is not only a complex multifactorial disease but also a metabolic disease [65]. Our study found that metabolic disorders in patients with AD may be associated with decreased HK activity because of the recruitment of HK by the LLPS of Tau-441. As a rate-limiting enzyme of glucose in the glycolysis pathway, a decrease of HK activity will cause the accumulation of glucose and lead to diabetes. Moreover, glucose, as the main energy source, cannot enter the glycolysis pathway to provide sufficient energy for cells, causing metabolic disorders of glucose, amino acids, and fatty acids in cells. Furthermore, in addition to the catalytic activity of the enzyme, HK is an effective pro-survival factor and can also antagonize apoptosis in the mitochondrial pathway. A decrease of HK activity can induce Bax-induced apoptosis. Thus, the decreased HK activity induced by Tau-441 LLPS plays an important role in apoptosis. In turn, HK in the solution drove Tau-441 LLPS. This puts Tau-441 LLPS and HK recruitment into a vicious circle. In our work, it was found that an MF (16 T) significantly inhibited the phase separation of Tau-441, which inhibited HK binding to Tau-441. Hence, the amount of HK competitively bound to VDAC I increased, leading to a decrease of VDAC I bound to Bax. In contrast, the induction of neuronal intrinsic apoptosis completely depends on the activation of Bax [66]. These results subsequently lead to a possible connection between Tau-441 phase separation and apoptosis. Furthermore, it has been reported that caspase-3/6 cleaved Tau at Asp421 to a more toxic form [67,68]. We have observed in this study that inhibition of caspase-3 activity with an MF (16 T) further reduced the deterioration of Tau and consequent cytotoxicity. Thus, an MF (16 T) can reduce cell apoptosis, which is rooted in the inhibition of Tau-441 LLPS. These inferences provide a theoretical basis for the possible application of MFs in clinical AD treatment.

In this study, we found that an MF (16 T) effectively inhibited the phase separation of Tau-441. This discovery suggested that we can make full use of this phenomenon in the treatment of LLPS-related diseases, such as AD as discussed above. In fact, interesting magnetobiological effects have always been compelling phenomena. However, for a long time, most of the phenomena have not been explained clearly by physical mechanisms, making these phenomena mysterious and difficult to understand, and some effects were even considered as pseudoscience. Using phase separation as a bridge for the first time, we found that an MF (16 T) can affect physiological or pathological processes by affecting the LLPS process, which provides an understandable explanation for the biological effect of MFs and also provides a theoretical basis for the use of MFs in the treatment of LLPS-related diseases.

MFs have great advantages as a potential physical therapy. First, MFs have strong tissue penetration. Most human tissues are diamagnetic. When an MF penetrates a tissue, there is almost no attenuation. This allows the MF to act on the deep tissues of the human body. Second, an MF is noninvasive and painless. The well-known repetitive transcranial magnetic stimulation technology takes full advantage of these features, which is now widely utilized in the clinical treatment of neurological diseases. MF in a suitable flux density range is safe for biological systems. The safety of the static MF (0 to 8 T) has been certified by the World Health Organization (https://www.who.int/publications/i/item/9241572329). This physical environment is harmless and even beneficial in most cases. Finally, permanent magnets can be used in magnetic therapy. We have studied the effect of a 0.48-T permanent magnet on the phase separation process of Tau-441. The results showed that, although the effect of 0.48 T was not as obvious as that of 16 T, the phase separation of Tau-441 was also effectively suppressed by 0.48 T (Fig. 2D to G). Therefore, it is feasible to use permanent magnets for practical magnetic therapy. Compared with the superconducting magnets, permanent magnets have the advantages of high simplicity, convenience, low cost, and no energy consumption.

This study provides a theoretical basis for the use of MFs to intervene in LLPS-related diseases. In the future, research on MF effects on LLPS can be further explored. New solutions may be found for the treatment of more LLPS-related diseases. In addition, the MF, as a representative of physical environments, demonstrated an influence on the LLPS process. Other physical environments, such as sound, light, electricity, force, and temperature, may also have potential effects on the LLPS process. Therefore, by studying the influence of these physical environments on the LLPS processes, it is expected that effective solutions to deal with LLPS-related diseases will be found.

## Materials and Methods

### MF application

The MF in this study was provided by a 16-T superconducting magnet (JMTA-16T, JASTEC Inc., Tokyo, Japan) [69]. Samples were placed into a sample holder and then inserted into the magnet bore; the temperature of which was controlled at 310.15 K by circulating temperature-controlled water from a water bath circulator (Polyscience 9712, Warrington, PA, USA) with a remote temperature sensor placed at the location of the sample [70].

The same environmental condition (sample holder inserted into a temperature-controlled cylindrical space) without the MF was also built and used for placing the control samples.

The 0.48-T MF in this study was provided by a permanent magnet apparatus [71]. The apparatus was a pair of cylindrical permanent magnets (NdFeB) with the south pole of one magnet facing right above the north pole of the other magnet. An identical control apparatus (also with a pair of cylindrical NdFeB objects, but not magnetized) was set next to the magnetic pair. An iron plate was placed between the 2 apparatuses to provide magnetic shielding. Both apparatuses were fixed into a sealed chamber, the temperature of which was controlled at 310.15 K by a circulating bath water using a water bath circulator (Polyscience 9712, USA). A flow of 5% CO_2_ was introduced into the chamber during experiments. Samples were placed into a sample holder, which was fixed between the permanent magnets. The magnetic field at the sample position was measured using a gauss meter (421 Gaussmeter, LakeShore Inc., Columbus, OH, USA).

A schematic illustration of the magnet systems and the magnetic field distribution are shown in Fig. S4.

### Expression and purification of recombinant Tau-441 protein

The expression and purification of recombinant Tau-441 protein were performed according to the method described as a supplementary method (see “Supporting method 1” in the Supplementary Materials). The highly purified Tau-441 protein was used for the LLPS solution experiments.

### Solution preparation and induction of LLPS

A Tau-441 protein solution (10 μM) was prepared by dissolving the protein into a buffer solution (150 mM NaCl, 10 mM 4-(2-hydroxyethyl)-1-piperazineethane-sulfonic acid (Hepes), 2 mM dithiothreitol, and 0.1 mM EDTA, pH 7.4); this buffer, hereafter called Hepes buffer in this paper, was used throughout the entire work. The solution was mixed with 10% PEG8000 to induce LLPS [25]. The solution was incubated at 310.15 K with and without MFs.

As a control, the native Tau-441 (10 μM) in the Hepes buffer solution was prepared and incubated at 310.15 K, without LLPS induction.

HK was purchased from Shanghai Yuanye Company (Shanghai Yuanye Bio-Technology Company, Shanghai, China) and prepared as a solution of 0.72 mg/ml with double-distilled water (ddH_2_O). Then, HK was mixed with the Tau-441 protein solution at a volume ratio of 1:9. After that, the mixed solutions with and without added 10% PEG8000 were placed inside and outside of the magnet at 310.15 K.

### Cross-linking of phase-separated droplets

The Tau-441 (10 μM) solution in Hepes buffer with 10% PEG8000 was incubated inside and outside MFs for 4 h. The phase-separated droplets formed in the solution were cross-linked and fixed by adding 1% glutaraldehyde [72] and incubated at 310.15 K for 6 h and then centrifuged at 5,000 g for 5 min. The supernatant was discarded, and the ions in the solution were removed by repeated washing with ddH_2_O. The fixed phase-separated droplets were examined using an optical microscope (Nikon ECLIPSE Ti2-E, Tokyo, Japan). The droplet sizes in the captured photos were analyzed by ImageJ software.

### Turbidity analysis

Tau-441 (10 μM) solutions in Hepes buffer and with different treatments were incubated at 310.15 K for 4 h. The OD_400_ (optical density at 400 nm) values of the samples at 200 μl were measured using an Epoch spectrophotometer (BioTek, Winooski, VT, USA) [14]. The turbidity of the Tau droplets in 200 μl of solution was measured using a 96-well plate.

### DLS analysis

Tau-441 (10 μM) solutions in Hepes buffer and with different treatments were incubated at 310.15 K for 4 h. DLS analyses were performed using a particle size analyzer (Zetasizer Nano ZS/ZEN3600, Malvern Instruments Limited, UK). The sizes were obtained from the statistical average of 4 independent experiments.

### CD spectroscopy analysis

Tau-441 (10 μM) solutions in Hepes buffer and with different treatments were incubated at 310.15 K for 4 h. The change of the secondary structure content of protein determined by CD was analyzed quantitatively. The spectropolarimeter (Chirascan, Applied Photophysics Ltd., Surrey, UK) was used to monitor the CD spectrum in the far ultraviolet range (200 to 260 nm). The experiment was repeated 3 times. DichroWeb software was used to deconvolve the spectra, and the results of secondary structure contents were obtained.

### Viscosity analysis of the Tau-441 protein solution

It is known that the viscosity is inversely proportional to the incidence of LLPS. To examine the effect of an MF (16 T) on solution viscosity, the Tau-441 protein (10 μM) solution with 10% PEG8000 was treated with and without an MF (16 T) for 24 h. An NJ-9S viscometer (Shanghai Pingxuan Science Instrument Co. Ltd., Shanghai, China) with a rotor was used to measure the viscosity of the Tau-441/PEG solution with the spindle number at 0 and the torque at 60 rpm.

### MF effect on the Tau-441 LLPS

Tau-441 (10 μM) in a buffer solution (150 mM NaCl and 10 mM Hepes (pH 7.4)) was incubated at 310.15 K for 24 h, with and without an MF (16 T).

A 10% PEG8000 solution was added to a Tau-441 (10 μM) solution in Hepes buffer to induce LLPS and incubated at 310.15 K for 24 h with and without MFs (0.48 and 16 T).

A Tau-441 (10 μM) solution was added to 10% PEG8000 in Hepes buffer to induce LLPS. After that, HK was added, and the mixed solutions were placed inside and outside the 16-T magnet at 310.15 K for 24 h. The change in the β-sheet content of the samples was measured on the basis of the fluorescence spectra.

ThT is a β-sheet binding dye and can label Tau-441 to monitor the LLPS reactions. ThT (0.8 mg/ml) was added to the sample solutions and incubated for 2 min. The fluorescence spectra (460 to 550 nm) were recorded with a fluorospectrophotometer (F-4500, Hitachi, Tokyo, Japan) with an excitation wavelength of 440 nm. The test samples with Tau-441 were incubated with and without an MF for 24 h.

### FTIR for studying the interaction between Tau-441 and HK

A Tau-441 (10 μM) solution was added to 10% PEG8000 in Hepes buffer to induce LLPS. After that, HK was added, and the mixed solutions were placed inside and outside the magnet (16 T) at 310.15 K for 24 h. The interaction between Tau-441 and HK was studied using the FTIR spectra in situ in real time.

The native Tau-441 (10 μM) in a buffer solution (150 mM NaCl and 10 mM Hepes (pH 7.4)) was prepared and incubated at 310.15 K. The FTIR spectra in situ in real time were used to study the change of native Tau-441.

A Vertex 70 spectrometer (Bruker Optics Inc., Ettinger, Germany) was used to measure the FTIR spectra of the samples. The spectral range from 500 to 4,000 cm^−1^ was recorded.

### AFM observation

The phase-separated solutions were diluted using ddH_2_O (18.3 MΩ) at the volume ratio 1:10,000. A drop of the diluted solution (1 μl) was dispensed on a polished silicon wafer using a pipette. The drop was blotted with a filter paper to remove excess solution and washed 3 times with ddH_2_O. After that, the samples were dried in air. Finally, the particles left on the silicon wafer were scanned using an AFM (Pico Plus 4500, Agilent, Santa Clara, CA, USA).

The imadjust function in MATLAB was used to process the AFM images. The linear mapping range was set to [255 × 0.4, 255 × 0.7] to remove the background noise, and the number of particles in the AFM images was statistically analyzed.

### Cell culture

293T and SK-N-SH cell lines were purchased from Kunming Cell Bank. The 293T cells were cultured in high-Dulbecco's modified Eagle's medium supplemented with 10% fetal bovine serum and 1% penicillin/streptomycin at 310.15 K and 5% CO_2_. The SK-N-SH cells were cultured in SK-N-SH cell-specific medium (iCell-h194-001b, iCellbioscience, Shanghai, China) at 310.15 K and 5% CO_2_.

### Construction of transgenic cell lines overexpressing Tau-441

293T cells were transfected with a plasmid containing the *MAPT* gene (encoding full-length human Tau) using Lipofectamine 2000 (Invitrogen, Carlsbad, CA, USA), according to the manufacturer’s instructions, and were incubated with 3 μg/ml of puromycin for 14 d. The selected monoclonal culture was verified with Western blotting. A lower dose of puromycin (1.5 μg/ml) was used for further cell culturing. The stably transfected cell line was named 293T-TAU441. 293T cells were used as a control.

SK-N-SH cells were transfected with a plasmid containing *MAPT* (full-length human Tau) using Lipo 6666 (MF769-02, Mei5 Biotechnology Co. Ltd, Beijing, China) according to the manufacturer’s instructions. The transfected cell line was named SK-N-SH-TAU441. SK-N-SH cells were used as a control.

### Arsenite induction of the Tau-441 LLPS in cultured cells and experimental grouping of cells

Cells were treated with 0.5 mM arsenite for 0.5 h to induce cellular LLPS, as previously reported [28].

Cells were divided into 2 groups: one group overexpressed *MAPT* and was treated with and without arsenite to induce LLPS and the other group was treated with 0.5 mM arsenite for 0.5 h and then cultured with and without an MF (16 T) for 6 or 24 h, with the original cells as controls.

### Caspase-3 activity assays

293T-TAU441 cells were incubated in 1 well of a 6-well plate. After culturing for 24 h, arsenite was added to the cells. Then, the cells were replaced with a fresh medium and placed inside and outside an MF (16 T) for 6 or 24 h. The activity of caspase-3 was quantified using a caspase-3 activity assay kit (C1115, Beyotime Biotechnology, Shanghai, China) according to the manufacturer’s instructions. The standard curve was established by using the *p*NA standard. The collected cells were lysed with buffer for 15 min and then centrifuged at 12,000 g for 15 min at 277.15 K. After collecting the supernatant, Ac-DEVD-*p*NA (2 mM) was added, and the lysate was incubated at 310.15 K for 2 h. The measurements were then performed using an enzyme labeling instrument at 405 nm (BioTek). The protein concentration was detected by the Bradford method.

### Cell apoptosis assays

Cell death was quantified using an Annexin V-PE apoptosis detection kit (C1065, Beyotime Biotechnology, China) according to the manufacturer’s instructions. The cells were centrifuged at 1,000 g for 5 min, the supernatant was discarded, 195 μl of Annexin V-PE binding solution was added, and the cells were gently resuspended. Finally, 5 μl of Annexin V-PE was added. The resulting aggregates were measured with a fluorescence spectrophotometer (F-4500, Hitachi, Japan) at 564-nm excitation and 575-nm emission wavelengths.

### Assessment of the cellular mitochondrial membrane potential

The changes of mitochondrial membrane potential were detected using a JC-1 mitochondrial membrane potential detection kit (M8650, Beijing Solarbio Science & Technology Co. Ltd, Beijing, China). Cells were treated with 0.5 mM arsenite for 0.5 h and then cultured with and without an MF (16 T). The cells were incubated with JC-1 at 310.15 K for 20 min. The aggregates represented by red fluorescence were measured with a fluorescence spectrophotometer (F-4500, Hitachi) at 585-nm excitation and 590-nm emission wavelengths, and the monomers represented by green fluorescence were measured at 515-nm excitation and 529-nm emission wavelengths.

### ROS analysis

The levels of intracellular ROS were analyzed by using a ROS assay kit (S0033, Beyotime Biotechnology, China). The 2',7'-dichlorodihydrofluorescein diacetate solutions were diluted using the culture medium at a volume ratio of 1:1,000. The cells were incubated in a cell incubator for 20 min at 310.15 K and 5% CO_2_. Then, the cells were washed 3 times with a medium. A fluorescence spectrophotometer (F-4500, Hitachi) was used to measure the intracellular fluorescence intensity. The excitation wavelength was 488 nm, and the emission wavelength was 525 nm. The experiment was conducted 3 times independently.

### Enzyme activity and compound content assays

After induction by arsenite for 0.5 h, the cells were cultured with and without an MF (16 T). The cells were washed 3 times in 1× phosphate-buffered saline. After trypsin digestion, the cells were lysed on ice for 30 min. After centrifuging the lysed cells at 2,000 g for 10 min, the supernatant was collected for enzyme activity and compound content analyses. The activity of HK was determined using commercial kits (A077-3-1, Hexokinase Assay Kit, Nanjing Jiancheng Bioengineering Institute, Nanjing, China). The concentrations of glucose 6-phosphate (G6P, S0185, Beyotime Biotechnology) and glucose (A154-2-1, Nanjing Jiancheng Bioengineering Institute) were also determined using commercial kits. The results were normalized to protein content.

### Co-IP assays

The cell supernatant was collected as in previous experiments and was quantified with the BCA method. Cell lysates were incubated with protein A/G magnetic beads (HY-K0202, MedChemExpress, Shanghai, China) combined with anti-Tau antibody (2 μg; sc-32274, Santa Cruz Biotechnology Inc., CA, USA), anti-VDAC I antibody (2 μg; AF1027, Beyotime Biotechnology), or immunoglobulin G (2 μg; 30000-0-AP, Proteintech, Wuhan, China) for 1 h at room temperature. The beads were washed and heat-denatured at 368.15 K for 5 min in sodium dodecyl sulfate polyacrylamide gel electrophoresis loading buffer before Western blotting analysis.

### Western blotting assays

The proteins were separated in a sodium dodecyl sulfate polyacrylamide gel electrophoresis gel and transferred onto a polyvinylidene fluoride membrane (Millipore, MA, USA) by wet transfer at 100 V. For immunodetection, the membrane was blocked for 1 h at room temperature with 5% bovine serum albumin, incubated with the primary antibody overnight at 277.15 K, and then incubated with the secondary antibody for 2 h at room temperature. The primary antibodies included anti-Tau (1:100; sc-32274, Santa Cruz Biotechnology Inc.), anti-HK1 (1:1,000; 19662-1-AP, Proteintech, Wuhan, China), anti-VDAC I (1:1,000; AF1027, Beyotime Biotechnology), anti-Bax (1:200; sc-7480, Santa Cruz Biotechnology Inc.), anti-PARP-1 (1:200; sc-8007, Santa Cruz Biotechnology Inc.), anti-caspase-3 (1:1,000; 9662S, Cell Signaling Technology, MA, USA), and anti-β-actin (E-AB-48018, Elabscience, Wuhan, China). The secondary antibodies included anti-mouse secondary antibody (HS201-01, TransGen Biotech, Beijing, China) at a 1:1,000 dilution or anti-rabbit secondary antibody (HS101-01, TransGen Biotech) at a 1:1,000 dilution. The signals were detected via chemiluminescence (Tanon, Shanghai, China).

### Homology modeling of Tau-441

The protein sequence of Tau-441 was obtained from the National Center for Biotechnology Information database. The homology model of the server-generated Tau-441 was constructed by the I-TASSER online protein modeler. I-TASSER is a template for automated homologous modeling. The server generates a large number of conformation sets in the initial stage of modeling. Then, the Spike program constructed on the I-TASSER server clustered all conformations according to their structural similarity. Finally, the server created 5 homologous models of Tau, which represented 5 structural clusters. The structures generated by I-TASSER were analyzed on the basis of the *C* score and the estimated TM score. A high value represents a high reliability model. The Tau models with the best *C* score, TM score, and clustering density were selected for further study.

### Molecular docking and calculation of binding energy

HK (1DGK), VADC I (6G73), and the MTBR domains of Tau-441 (7P6B) were selected from the PDB (RCSB Protein Data Bank; https://www.rcsb.org/) database. PyMOL software was used to remove water, glucose, ADP, and phosphate ions from HK. The VDAC I and MTBR domains of the Tau-441 polymer were decomposed into monomers using the same software. ZDOCK online software was used to analyze molecular docking. ZDOCK software generated the top 10 predictions according to the free energy gains. Amino acid interactions (hydrogen bonds and hydrophobic interactions) in the protein–protein interactions were analyzed using LigPlot software. The solvation free energy gain was calculated using PDBePISA online software.

### Statistical analysis

All data in this study were expressed as means ± SEM. Differences between 2 groups were analyzed by Student’s unpaired *t* tests. Differences between multiple groups were analyzed by analysis of variance. Differences were considered as statistically significant when *P* < 0.05.

## Data Availability

All data needed to evaluate the conclusions in the paper are present in the paper and the Supplementary Materials. Additional data related to this paper may be requested from the authors.

## Supplementary Materials

Supplementary Materials Methods, measurements, figures, and tables. Fig. S1. Putative binding sites between Tau-441 and hexokinase (HK). Fig. S2. Putative binding sites between homologous modeling of native Tau-441 and HK. Fig. S3. Binding sites of recombinant human HK type I with ADP, phosphate ion, and glucose. Fig. S4. Schematic illustration and magnetic field distribution of the magnets. Fig. S5. Fourier transform infrared spectroscopy (FTIR) spectra of native Tau-441 solution at 310.15 K. Fig. S6. Putative binding sites between voltage-dependent anion channel (VDAC I) and HK. Fig. S7. Apoptosis of 293T-TAU441 cells was decreased by an MF (16 T) induction for 6 h. Fig. S8. Apoptosis of SK-N-SH-TAU441 cells was decreased by an MF (16 T) induction for 6 h. Fig. S9. Apoptosis of SK-N-SH-TAU441 cells was decreased by an MF (16 T) induction for 24 h. Table S1. Top 10 possible solvation free energy gains upon HK binding to native Tau-441 calculated by the molecular docking method. Table S2. Top 10 possible solvation free energy gains upon HK binding to VDAC calculated by the molecular docking method.

## References

[1] Chiu K-H, Ou K-L, Lee S-Y, Lin CT, Chang WJ, Chen CC, Huang HM. Static magnetic fields promote osteoblast-like cells differentiation via increasing the membrane rigidity. Ann Biomed Eng. 2007;35(11):1932–1939. 17721730 10.1007/s10439-007-9370-2

[2] Bobkova NV, Novikov VV, Medvinskaya NI, Aleksandrova IY, Nesterova IV, Fesenko EE. Effect of weak combined static and extremely low-frequency alternating magnetic fields on spatial memory and brain amyloid-β in two animal models of Alzheimer’s disease. Electromagn Biol Med. 2018;37(3):127–137. 29771571 10.1080/15368378.2018.1471700

[3] Zhang B, Wang L, Zhan A, Wang M, Tian L, Guo W, Pan Y. Long-term exposure to a hypomagnetic field attenuates adult hippocampal neurogenesis and cognition. Nat Commun. 2021;12(1):1174–1191. 33608552 10.1038/s41467-021-21468-x PMC7896063

[4] Leon-Sarmiento FE, Gonzalez-Castaño A, Rizzo-Sierra CV, Aceros J, Leon-Ariza DS, Leon-Ariza JS, Prada DG, Bara-Jimenez W, Wang ZY. Neurophysics assessment of the muscle bioenergy generated by transcranial magnetic stimulation. Research. 2019;2019:1–9. 10.34133/2019/7109535 PMC6750091 31549082

[5] Fox MD, Buckner RL, Liu H, Chakravarty MM, Lozano AM, Pascual-Leone A. Resting-state networks link invasive and noninvasive brain stimulation across diverse psychiatric and neurological diseases. Proc Natl Acad Sci USA. 2014;111(41):E4367–E4375. 25267639 10.1073/pnas.1405003111 PMC4205651

[6] Langa KM. Is the risk of Alzheimer’s disease and dementia declining? Alzheimers Res Ther. 2015;7(1):34–38. 25815064 10.1186/s13195-015-0118-1 PMC4374373

[7] Wong W. Economic burden of Alzheimer disease and managed care considerations. Am J Manag Care. 2020;26(8 Suppl):S177–S183. 32840331 10.37765/ajmc.2020.88482

[8] Butterfield DA, Halliwell B. Oxidative stress, dysfunctional glucose metabolism and Alzheimer disease. Nat Rev Neurosci. 2019;20(3):148–160. 30737462 10.1038/s41583-019-0132-6 PMC9382875

[9] Arendt T, Brückner MK, Morawski M, Jäger C, Gertz H-J. Early neurone loss in Alzheimer’s disease: Cortical or subcortical? Acta Neuropathol Commun. 2015;3: Article 10. 10.1186/s40478-015-0187-1 PMC4359478 25853173

[10] Buée L, Troquier L, Burnouf S, Belarbi K, van der Jeugd A, Ahmed T, Fernandez-Gomez F, Caillierez R, Grosjean ME, Begard S, et al. From tau phosphorylation to tau aggregation: What about neuronal death? Biochem Soc Trans. 2010;38(4):967–972. 20658986 10.1042/BST0380967

[11] Kraemer BC, Zhang B, Leverenz JB, Thomas JH, Trojanowski JQ, Schellenberg GD. Neurodegeneration and defective neurotransmission in a Caenorhabditis elegans model of tauopathy. Proc Natl Acad Sci USA. 2003;100(17):9980–9985. 12872001 10.1073/pnas.1533448100 PMC187908

[12] Olsson B, Lautner R, Andreasson U, Öhrfelt A, Portelius E, Bjerke M, Hölttä M, Rosén C, Olsson C, Strobel G, et al. CSF and blood biomarkers for the diagnosis of Alzheimer’s disease: A systematic review and meta-analysis. Lancet Neurol. 2016;15(7):673–684. 27068280 10.1016/S1474-4422(16)00070-3

[13] Wegmann S, Medalsy ID, Mandelkow E, Müller DJ. The fuzzy coat of pathological human Tau fibrils is a two-layered polyelectrolyte brush. Proc Natl Acad Sci USA. 2013;110(4):E313–E321. 23269837 10.1073/pnas.1212100110 PMC3557036

[14] Boyko S, Qi X, Chen T-H, Surewicz K, Surewicz WK. Liquid-liquid phase separation of tau protein: The crucial role of electrostatic interactions. J Biol Chem. 2019;294(29):11054–11059. 31097543 10.1074/jbc.AC119.009198 PMC6643045

[15] Brangwynne CP, Eckmann CR, Courson DS, Rybarska A, Hoege C, Gharakhani J, Jülicher F, Hyman AA. Germline P granules are liquid droplets that localize by controlled dissolution/condensation. Science. 2009;324(5935):1729–1732. 19460965 10.1126/science.1172046

[16] Sabari BR, Dall'Agnese A, Boija A, Klein IA, Coffey EL, Shrinivas K, Abraham BJ, Hannett NM, Zamudio AV, Manteiga JC, et al. Coactivator condensation at super-enhancers links phase separation and gene control. Science. 2018;361(6400): eaar3958. 29930091 10.1126/science.aar3958 PMC6092193

[17] Patel A, Lee HO, Jawerth L, Maharana S, Jahnel M, Hein MY, Stoynov S, Mahamid J, Saha S, Franzmann TM, et al. A liquid-to-solid phase transition of the ALS protein FUS accelerated by disease mutation. Cell. 2015;162(5):1066–1077. 26317470 10.1016/j.cell.2015.07.047

[18] Fujioka Y, Alam JM, Noshiro D, Mouri K, Ando T, Okada Y, May AI, Knorr RL, Suzuki K, Ohsumi Y, et al. Phase separation organizes the site of autophagosome formation. Nature. 2020;578(7794):301–305. 32025038 10.1038/s41586-020-1977-6

[19] Setru SU, Gouveia B, Alfaro-Aco R, Shaevitz JW, Stone HA, Petry S. A hydrodynamic instability drives protein droplet formation on microtubules to nucleate branches. Nat Phys. 2021;17(4):493–498. 35211183 10.1038/s41567-020-01141-8 PMC8865447

[20] Quail T, Golfier S, Elsner M, Ishihara K, Murugesan V, Renger R, Jülicher F, Brugués J. Force generation by protein–DNA co-condensation. Nat Phys. 2021;17(9):1007–1012.

[21] Klosin A, Oltsch F, Harmon T, Honigmann A, Jülicher F, Hyman AA, Zechner C. Phase separation provides a mechanism to reduce noise in cells. Science. 2020;367(6476):464–468. 31974256 10.1126/science.aav6691

[22] Lei Z, Wang L, Kim EY, Cho J. Phase separation of chromatin and small RNA pathways in plants. Plant J. 2021;108(5):1256–1265. 34585805 10.1111/tpj.15517

[23] Banani SF, Lee HO, Hyman AA, Rosen MK. Biomolecular condensates: Organizers of cellular biochemistry. Nat Rev Mol Cell Biol. 2017;18(5):285–298. 28225081 10.1038/nrm.2017.7 PMC7434221

[24] Zhang Y, Fan S, Hua C, Teo ZWN, Kiang JX, Shen L, Yu H. Phase separation of HRLP regulates flowering time in *Arabidopsis*. Sci Adv. 2022;8(25): Article eabn5488. 35731874 10.1126/sciadv.abn5488 PMC9217094

[25] Kanaan NM, Hamel C, Grabinski T, Combs B. Liquid-liquid phase separation induces pathogenic tau conformations in vitro. Nat Commun. 2020;11(1):2809–2825. 32499559 10.1038/s41467-020-16580-3 PMC7272632

[26] Ray S, Singh N, Kumar R, Patel K, Pandey S, Datta D, Mahato J, Panigrahi R, Navalkar A, Mehra S, et al. α-Synuclein aggregation nucleates through liquid-liquid phase separation. Nat Chem. 2020;12(8):705–716. 32514159 10.1038/s41557-020-0465-9

[27] Birsa N, Ule AM, Garone MG, Tsang B, Mattedi F, Chong PA, Humphrey J, Jarvis S, Pisiren M, Wilkins OG, et al. FUS-ALS mutants alter FMRP phase separation equilibrium and impair protein translation. Sci Adv. 2021;7(30): Article eabf8660. 34290090 10.1126/sciadv.abf8660 PMC8294762

[28] Vanderweyde T, Apicco DJ, Youmans-Kidder K, Ash PEA, Cook C, Lummertz da Rocha E, Jansen-West K, Frame AA, Citro A, Leszyk JD, et al. Interaction of tau with the RNA-binding protein TIA1 regulates tau pathophysiology and toxicity. Cell Rep. 2016;15(7):1455–1466. 27160897 10.1016/j.celrep.2016.04.045 PMC5325702

[29] Hashimoto S, Matsuba Y, Kamano N, Mihira N, Sahara N, Takano J, Muramatsu SI, Saido TC, Saito T. Tau binding protein CAPON induces tau aggregation and neurodegeneration. Nat Commun. 2019;10(1):2394–2410. 31160584 10.1038/s41467-019-10278-x PMC6546774

[30] Stefanoska K, Volkerling A, Bertz J, Poljak A, Ke YD, Ittner LM, Ittner A. An N-terminal motif unique to primate tau enables differential protein-protein interactions. J Biol Chem. 2018;293(10):3710–3719. 29382714 10.1074/jbc.RA118.001784 PMC5846163

[31] Agostini M, Romeo F, Inoue S, Niklison-Chirou MV, Elia AJ, Dinsdale D, Morone N, Knight RA, Mak TW, Melino G. Metabolic reprogramming during neuronal differentiation. Cell Death Differ. 2016;23(9):1502–1514. 27058317 10.1038/cdd.2016.36 PMC5072427

[32] Schindler A, Foley E. Hexokinase 1 blocks apoptotic signals at the mitochondria. Cell Signal. 2013;25(12):2685–2692. 24018046 10.1016/j.cellsig.2013.08.035

[33] Robey RB, Hay N. Mitochondrial hexokinases: Guardians of the mitochondria. Cell Cycle. 2005;4(5):654–658. 15846094 10.4161/cc.4.5.1678

[34] Harris RA, Tindale L, Cumming RC. Age-dependent metabolic dysregulation in cancer and Alzheimer’s disease. Biogerontology. 2014;15(6):559–577. 25305052 10.1007/s10522-014-9534-z

[35] Lin Y, Fichou Y, Longhini AP, Llanes LC, Yin P, Bazan GC, Kosik KS, Han S. Liquid-liquid phase separation of tau driven by hydrophobic interaction facilitates fibrillization of tau. J Mol Biol. 2021;433(2): 166731. 33279579 10.1016/j.jmb.2020.166731 PMC7855949

[36] Alberti S, Gladfelter A, Mittag T. Considerations and challenges in studying liquid-liquid phase separation and biomolecular condensates. Cell. 2019;176(3):419–434. 30682370 10.1016/j.cell.2018.12.035 PMC6445271

[37] Wang F, Altschuh P, Ratke L, Zhang H, Selzer M, Nestler B. Progress report on phase separation in polymer solutions. Adv Mater. 2019;31(26):1806733–1806747. 10.1002/adma.201806733 30856293

[38] Feng Y, Lin W, Murillo MS. Viscosity of two-dimensional strongly coupled dusty plasma modified by a perpendicular magnetic field. Phys Rev E. 2017;96(5): Article 053208. 29347770 10.1103/PhysRevE.96.053208

[39] Liu Y-M, Wu Z-Q, Bao S, Guo WH, Li DW, He J, Zeng XB, Huang LJ, Lu QQ, Guo YZ, et al. The possibility of changing the wettability of material surface by adjusting gravity. Research. 2020;2020: Article 2640834. 32043083 10.34133/2020/2640834 PMC7007757

[40] Aleshin AE, Kirby C, Liu X, Bourenkov GP, Bartunik HD, Fromm HJ, Honzatko RB. Crystal structures of mutant monomeric hexokinase I reveal multiple ADP binding sites and conformational changes relevant to allosteric regulation. J Mol Biol. 2000;296(4):1001–1015. 10686099 10.1006/jmbi.1999.3494

[41] Shi Y, Zhang W, Yang Y, Murzin AG, Falcon B, Kotecha A, van Beers M, Tarutani A, Kametani F, Garringer HJ, et al. Structure-based classification of tauopathies. Nature. 2021;598(7880):359–363. 34588692 10.1038/s41586-021-03911-7 PMC7611841

[42] Pierce BG, Wiehe K, Hwang H, Kim BH, Vreven T, Weng Z. ZDOCK server: Interactive docking prediction of protein-protein complexes and symmetric multimers. Bioinformatics. 2014;30(12):1771–1773. 24532726 10.1093/bioinformatics/btu097 PMC4058926

[43] Wallace AC, Laskowski RA, Thornton JM. LIGPLOT: A program to generate schematic diagrams of protein-ligand interactions. Protein Eng. 1995;8(2):127–134. 7630882 10.1093/protein/8.2.127

[44] Zheng W, Zhang C, Li Y, Pearce R, Bell EW, Zhang Y. Folding non-homologous proteins by coupling deep-learning contact maps with I-TASSER assembly simulations. Cell Rep Methods. 2021;1(3):100014–100039. 34355210 10.1016/j.crmeth.2021.100014 PMC8336924

[45] Krissinel E, Henrick K. Inference of macromolecular assemblies from crystalline state. J Mol Biol. 2007;372(3):774–797. 17681537 10.1016/j.jmb.2007.05.022

[46] Liu X, Zhou T, Wang X, Zhang J. Investigation of selective molecular interactions using two-dimensional Fourier transform IR spectroscopy. Anal Bioanal Chem. 2010;397(1):339–343. 20084374 10.1007/s00216-009-3403-7

[47] Sip S, Rosiak N, Miklaszewski A, Talarska P, Dudziec E, Cielecka-Piontek J. Amorphous form of carvedilol phosphate-the case of divergent properties. Molecules. 2021;26(17):5318–5333. 34500748 10.3390/molecules26175318 PMC8434513

[48] Zojaji I, Esfandiarian A, Taheri-Shakib J. Toward molecular characterization of asphaltene from different origins under different conditions by means of FT-IR spectroscopy. Adv Colloid Interf Sci. 2021;289: Article 102314. 10.1016/j.cis.2020.102314 33561569

[49] Pyne P, Mitra RK. Excipients do regulate phase separation in lysozyme and thus also its hydration. J Phys Chem Lett. 2022;13(3):931–938. 35050625 10.1021/acs.jpclett.1c03449

[50] Khan A, Wang C, Sun X, Killpartrick A, Guo M. Physicochemical and microstructural properties of polymerized whey protein encapsulated 3,3’-diindolylmethane nanoparticles. Molecules. 2019;24(4):702–718. 30781356 10.3390/molecules24040702 PMC6412796

[51] Shoshan-Barmatz V, Zakar M, Rosenthal K, Abu-Hamad S. Key regions of VDAC1 functioning in apoptosis induction and regulation by hexokinase. Biochim Biophys Acta. 2009;1787(5):421–430. 19094960 10.1016/j.bbabio.2008.11.009

[52] Tang Q, Bai L, Zou Z, Meng P, Xia Y, Cheng S, Mu S, Zhou J, Wang X, Qin X, et al. Ferroptosis is newly characterized form of neuronal cell death in response to arsenite exposure. Neurotoxicology. 2018;67:27–36. 29678591 10.1016/j.neuro.2018.04.012

[53] Yang W, Yue H, Lu G, Wang W, Deng Y, Ma G, Wei W. Advances in delivering oxidative modulators for disease therapy. Research. 2022;2022: Article 9897464. 10.34133/2022/9897464 PMC11278358 39070608

[54] Yamaguchi M, Tanimoto Y. *Magneto-Science*. Berlin (Germany): Springer; 2006. Fundamentals of magnetic field effects; p. 1–40.

[55] Von Bergen M, Barghorn S, Jeganathan S, Mandelkow E-M, Mandelkow E. Spectroscopic approaches to the conformation of tau protein in solution and in paired helical filaments. Neurodegener Dis. 2006;3(4–5):197–206. 17047358 10.1159/000095257

[56] Li T, Kheifets S, Medellin D, Raizen MG. Measurement of the instantaneous velocity of a Brownian particle. Science. 2010;328(5986):1673–1675. 20488989 10.1126/science.1189403

[57] Yin DC, Inatomi Y, Wakayama NI, Huang WD, Kuribayashi K. An investigation of magnetic field effects on the dissolution of lysozyme crystal and related phenomena. Acta Crystallogr D. 2002;58(12):2024–2030. 12454460 10.1107/s0907444902015524

[58] Zhong C, Wakayama NI. Effect of a high magnetic field on the viscosity of an aqueous solution of protein. J Cryst Growth. 2001;226(2–3):327–332.

[59] Munder T, Pfeffer A, Schreyer S, Guo J, Braun J, Sack I, Steiner B, Klein C. MR elastography detection of early viscoelastic response of the murine hippocampus to amyloid β accumulation and neuronal cell loss due to Alzheimer’s disease. J Magn Reson Imaging. 2018;47(1):105–114. 28422391 10.1002/jmri.25741

[60] Sarkar A, Messerli MA. Electrokinetic perfusion through three-dimensional culture reduces cell mortality. Tissue Eng Part A. 2021;27(23–24):1470–1479. 33820474 10.1089/ten.TEA.2021.0008

[61] Radvar E, Shi Y, Grasso S, Edwards-Gayle CJC, Liu X, Mauter MS, Castelletto V, Hamley IW, Reece MJ, S. Azevedo H. Magnetic field-induced alignment of nanofibrous supramolecular membranes: A molecular design approach to create tissue-like biomaterials. ACS Appl Mater Interfaces. 2020;12(20):22661–22672. 32283011 10.1021/acsami.0c05191

[62] Han Y, Cao Y, Zhou J, Yao Y, Wu X, Bolisetty S, Diener M, Handschin S, Lu C, Mezzenga R. Interfacial electrostatic self-assembly of amyloid fibrils into multifunctional protein films. Adv Sci. 2023;10(9): Article 2206867. 10.1002/advs.202206867 PMC10037951 36698306

[63] Saijo S, Yamada Y, Sato T, Tanaka N, Matsui T, Sazaki G, Nakajima K, Matsuura Y. Structural consequences of hen egg-white lysozyme orthorhombic crystal growth in a high magnetic field: Validation of X-ray diffraction intensity, conformational energy searching and quantitative analysis of B factors and mosaicity. Acta Crystallogr D Biol Crystallogr. 2005;61(Pt 3):207–217. 15735330 10.1107/S0907444904030926

[64] Yin D-C. Protein crystallization in a magnetic field. Prog Cryst Growth Charact Mater. 2015;61(1):1–26.

[65] Demetrius LA, Driver J. Alzheimer’s as a metabolic disease. Biogerontology. 2013;14(6):641–649. 24249045 10.1007/s10522-013-9479-7

[66] Fricker M, Tolkovsky AM, Borutaite V, Coleman M, Brown GC. Neuronal cell death. Physiol Rev. 2018;98(2):813–880. 29488822 10.1152/physrev.00011.2017 PMC5966715

[67] Rissman RA, Poon WW, Blurton-Jones M, Oddo S, Torp R, Vitek MP, LaFerla FM, Rohn TT, Cotman CW. Caspase-cleavage of tau is an early event in Alzheimer disease tangle pathology. J Clin Invest. 2004;114(1):121–130. 15232619 10.1172/JCI20640 PMC437967

[68] Gamblin TC, Chen F, Zambrano A, Abraha A, Lagalwar S, Guillozet AL, Lu M, Fu Y, Garcia-Sierra F, LaPointe N, et al. Caspase cleavage of tau: Linking amyloid and neurofibrillary tangles in Alzheimer’s disease. Proc Natl Acad Sci USA. 2003;100(17):10032–10037. 12888622 10.1073/pnas.1630428100 PMC187753

[69] Yan E-K, Zhang C-Y, He J, Yin DC. An overview of hardware for protein crystallization in a magnetic field. Int J Mol Sci. 2016;17(11):1906–1925. 27854318 10.3390/ijms17111906 PMC5133904

[70] Lu H-M, Yin D-C, Li H-S, Geng LQ, Zhang CY, Lu QQ, Guo YZ, Guo WH, Shang P, Wakayama NI. A containerless levitation setup for liquid processing in a superconducting magnet. Rev Sci Instrum. 2008;79(9):093903–093911. 19044425 10.1063/1.2980383

[71] Liu Y-L, Li D-W, He J, Xie XZ, Chen D, Yan EK, Ye YJ, Yin DC. A periodic magnetic field as a special environment for scientific research created by rotating permanent magnet pairs. Rev Sci Instrum. 2018;89(10): Article 105103. 30399658 10.1063/1.5016570

[72] Yan E-K, Lu Q-Q, Zhang C-Y, Liu YL, He J, Chen D, Wang B, Zhou RB, Wu P, Yin DC. Preparation of cross-linked hen-egg white lysozyme crystals free of cracks. Sci Rep. 2016;6:34770–34779. 27703210 10.1038/srep34770 PMC5050519

