# Supplementary material for: Magnetic Fields Reduce Apoptosis by Suppressing Phase Separation of Tau-441
Source: Research (Wash D C). 2023 May 11;6:0146. doi: 10.34133/research.0146 (PMC10204748; doi:10.34133/research.0146)
Supplement: Supplementary Materials — Methods, measurements, figures, and tables. Fig. S1. Putative binding sites between Tau-441 and hexokinase (HK). Fig. S2. Putative binding sites between homologous modeling of native Tau-441 and HK. Fig. S3. Binding sites of recombinant human HK type I with ADP, phosphate ion, and glucose. Fig. S4. Schematic illustration and magnetic field distribution of the magnets. Fig. S5. Fourier transform infrared spectroscopy (FTIR) spectra of native Tau-441 solution at 310.15 K. Fig. S6. Putative binding sites between voltage-dependent anion channel (VDAC I) and HK. Fig. S7. Apoptosis of 293T-TAU441 cells was decreased by an MF (16 T) induction for 6 h. Fig. S8. Apoptosis of SK-N-SH-TAU441 cells was decreased by an MF (16 T) induction for 6 h. Fig. S9. Apoptosis of SK-N-SH-TAU441 cells was decreased by an MF (16 T) induction for 24 h. Table S1. Top 10 possible solvation free energy gains upon HK binding to native Tau-441 calculated by the molecular docking method. Table S2. Top 10 possible solvation free energy gains upon HK binding to VDAC calculated by the molecular docking method. [file research.0146.f1.docx]

Magnetic Fields Reduce Apoptosis by Suppressing Phase Separation of Tau-441

**Magnetic Fields Affect Biosystems Through Phase Separation**

Wen-Juan Lin^†^, Wen-Pu Shi^†^, Wan-Yi Ge, Liang-Liang Chen, Wei-Hong Guo, Peng Shang, Da-Chuan Yin*

Key Laboratory for Space Bioscience and Biotechnology, School of Life Sciences, Northwestern Polytechnical University, 127 Youyixi Road, Xi'an 710072, Shaanxi, PR China.

*Corresponding author name: Da-Chuan Yin.

Email: [yindc@nwpu.edu.cn](mailto:yindc@nwpu.edu.cn).

ORCID: 0000-0003-2161-5867.

†These authors contributed equally to this work.

**Supporting Information Text**

**Supporting Methods**

**Supporting method 1: Expression and purification of recombinant Tau-441 protein**

Tau-441 protein used in this study contains 441 amino acids and predominantly found in Alzheimer’s disease. The protein was expressed in an *E. coli* strain and purified as minor modification on the basis of previous research [1]. The bacteria were incubated in Luria Broth (LB) at 310.15K until the medium was at OD_600_ = 0.6-0.8. IPTG at 1 mM was added to the culture which incubated for additional 4 hrs. Cells were homogenized in lysis buffer (50 mM NaH_2_PO_4_ pH = 8, 300 mM NaCl, 10 mM imidazole plus 2 mM PMSF, 1 mg/mL lysozyme). The protein was incubated in Ni NTA Agarose (Lanxiao, Shaanxi, China). The protein was added to a purification column (Lanxiao, Shaanxi, China) which was then washed with NaH_2_PO_4_ buffer (20 mM imidazole) for three times, and eluted with 250 mM imidazole. The obtained protein was quantified by SDS-PAGE and Western blotting.

The purity and qualitative analysis of purified Tau-441 protein were performed by SDS-PAGE and Western blotting.

**Supporting method 2: Derivation of the difference in magnetic susceptibility *(Δ****χ***)** **between phase separated droplet and surrounding bulk solution**

A droplet in a gradient magnetic field will subject to a magnetic force *F*:

**$F=-V\frac{\chi}{\mu_{0}}BB'$**  **(1)**

where *V* is the volume of the droplet, *χ* is the volume magnetic susceptibility, *μ*_0_ is the magnetic permeability in vacuum (which is a constant), B and B′ are the magnetic field and its gradient.

Under the magnetic field condition, when *F* = *ρVg* the droplet will be levitated:

  $\text{-}V\frac{\chi}{\mu_{0}}BB'=\rho Vg$ **(2)**

where *ρ* is the density of the droplet, *V* is the volume of the droplet, *g* is the acceleration of gravity.

According to Equation (2), we can obtain *Δχ* (which is the difference in the magnetic susceptibility between the phase-separated droplet and the surrounding bulk solution):

  $\Delta\chi=\frac{\mu_{0}\rho_{1}\text{g}}{\left(BB'\right)_{1}}-\frac{\mu_{0}\rho_{2}g}{\left(BB'\right)_{2}}$ **(3)**

where *ρ*_1_ is the density of the surrounding bulk solution, *ρ*_2_ is the density of the phase-separated droplet, *g* is the acceleration of gravity. (BB′)_1_ and (BB′)_2_ are the products of the magnetic field and its gradient at the positions the bulk solution and phase separated droplet levitated.

We levitated the bulk solution and phase separated droplet in the superconducting magnet, and recorded the levitation positions (at 352 mm and 354mm from the bottom of the magnet, respectively). According to the positions, (BB′)_1_ and (BB′)_2_ can be obtained from the superconducting datasheet. Hence the difference in the magnetic susceptibility *Δχ* can be obtained. Here the result is: *Δχ =* 1.2×10^-6^*.*

**Supporting Figures**


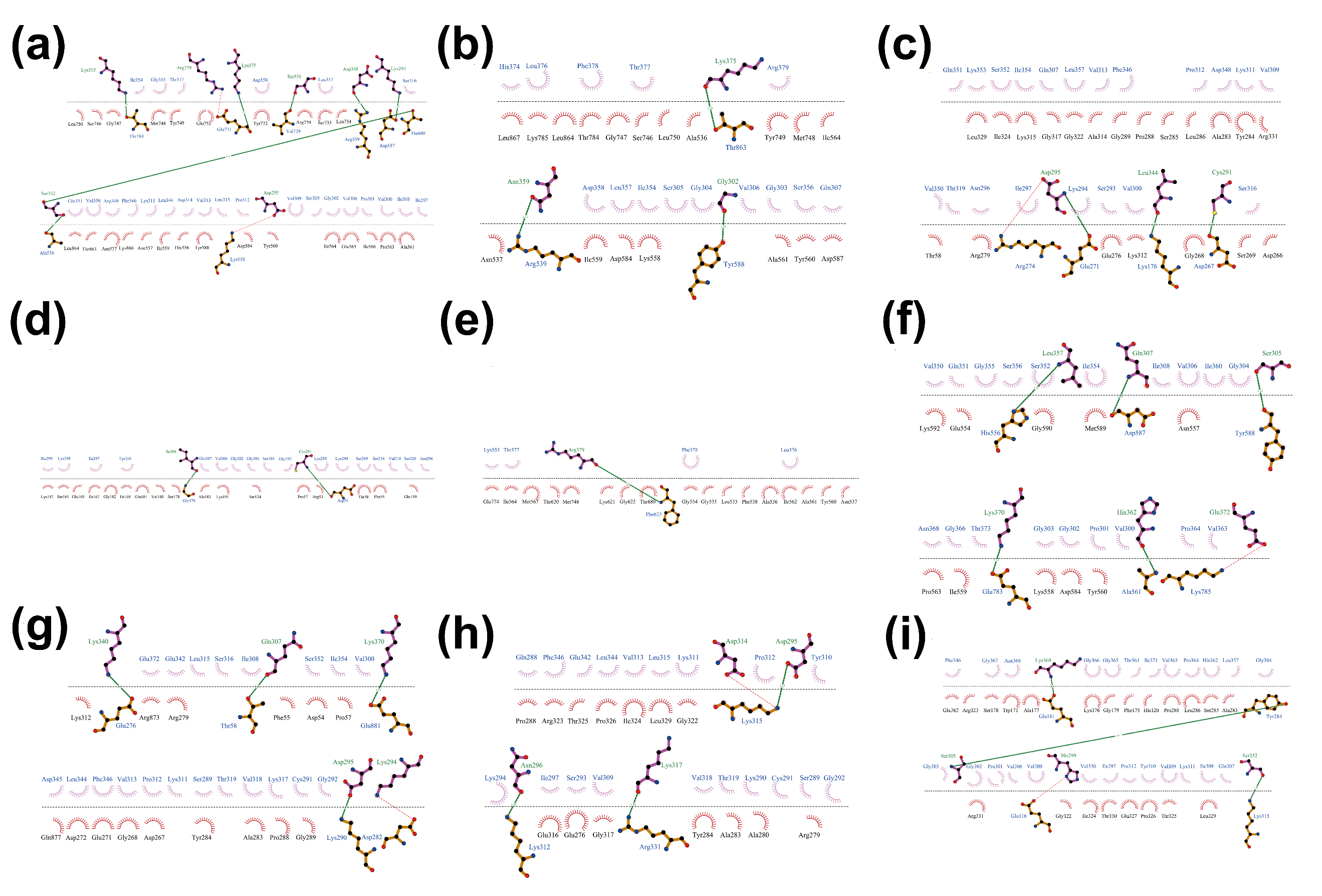


**Figure S1. Putative binding sites between Tau-441 and** **hexokinase (HK).** The top nine possible binding sites of HK binding to the Tau-441 MTB region shown by ZDOCK and Ligplot software. The dotted green line indicates hydrogen bonds, and the arc with spokes radiating toward the ligand atoms indicates hydrophobic interactions.


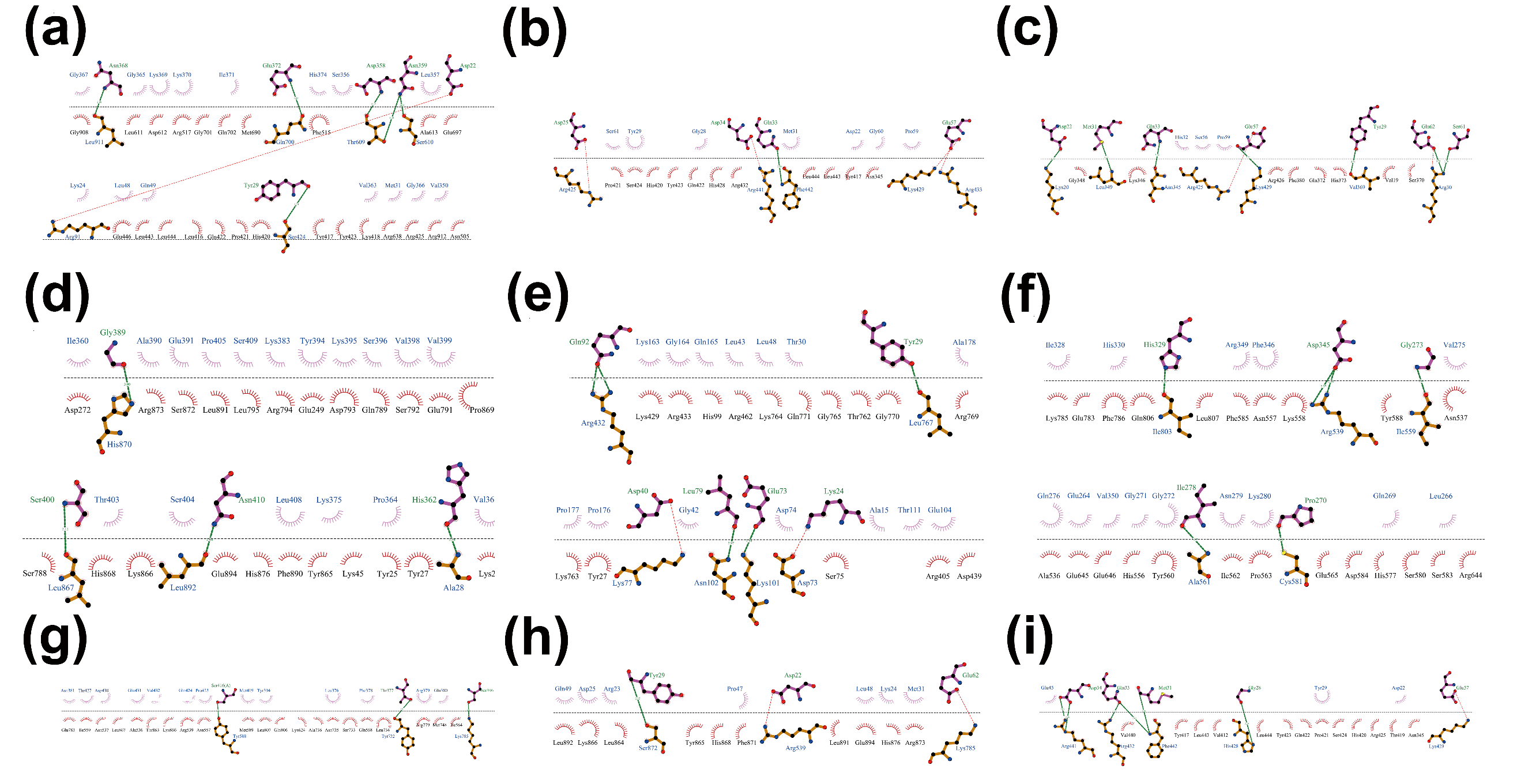


**Figure S2. Putative binding sites between homologous modeling of native Tau-441 and hexokinase (HK).** The top nine possible binding sites of HK binding to homologous modeling of native Tau-441 shown by ZDOCK and Ligplot software. The dotted green line indicates hydrogen bonds, and the arc with spokes radiating toward the ligand atoms indicates hydrophobic interactions.


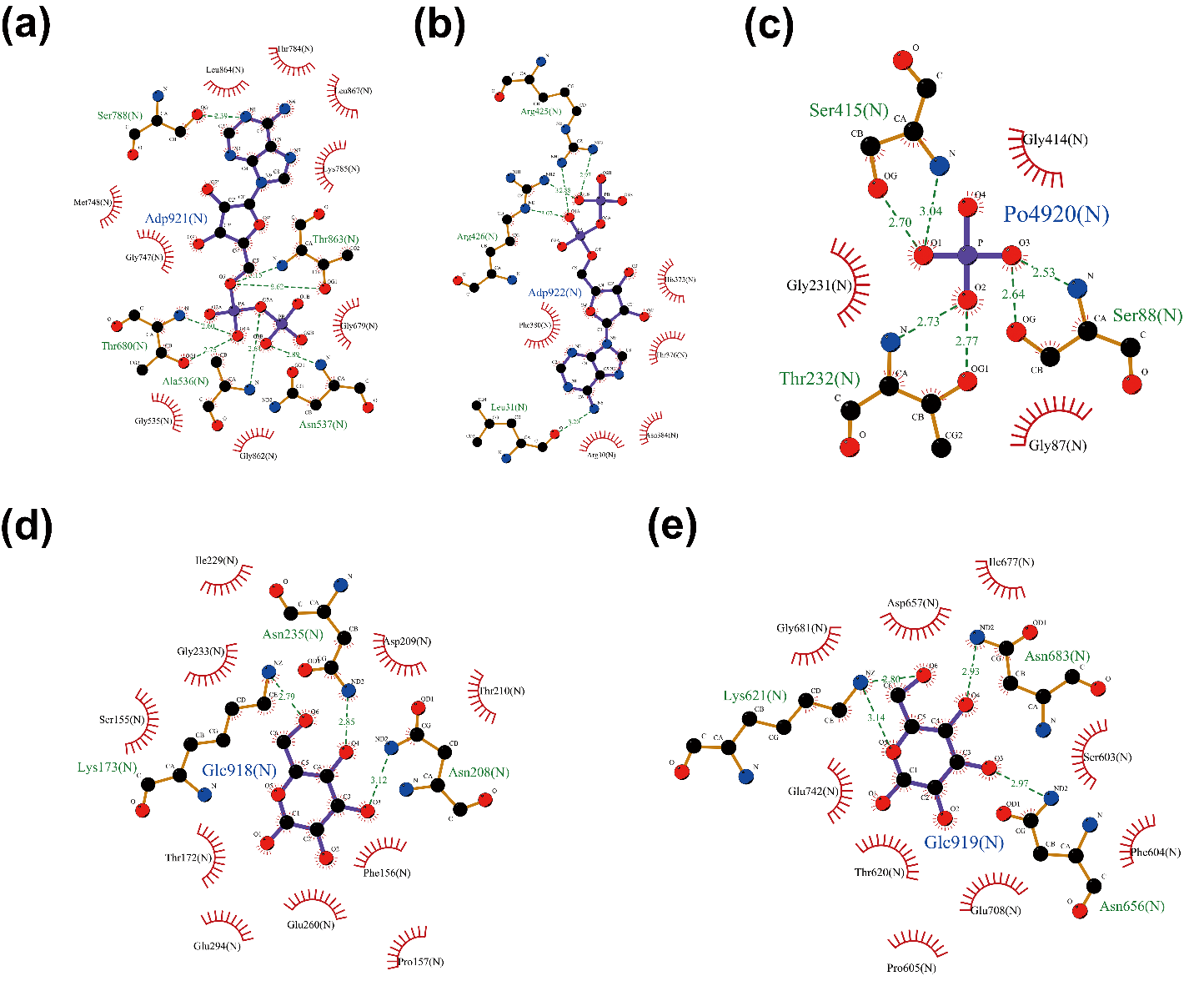


**Figure S3. Binding sites of recombinant human hexokinase type I with ADP, phosphate ion, and glucose.** (a), (b) Binding sites of hexokinase (HK) with ADP. (c) Binding sites of HK with phosphate ion. (d), (e) Binding sites of HK with glucose. The dotted green lines indicate hydrogen bonds, and the arc with spokes radiating toward the ligand atoms indicates hydrophobic interactions.


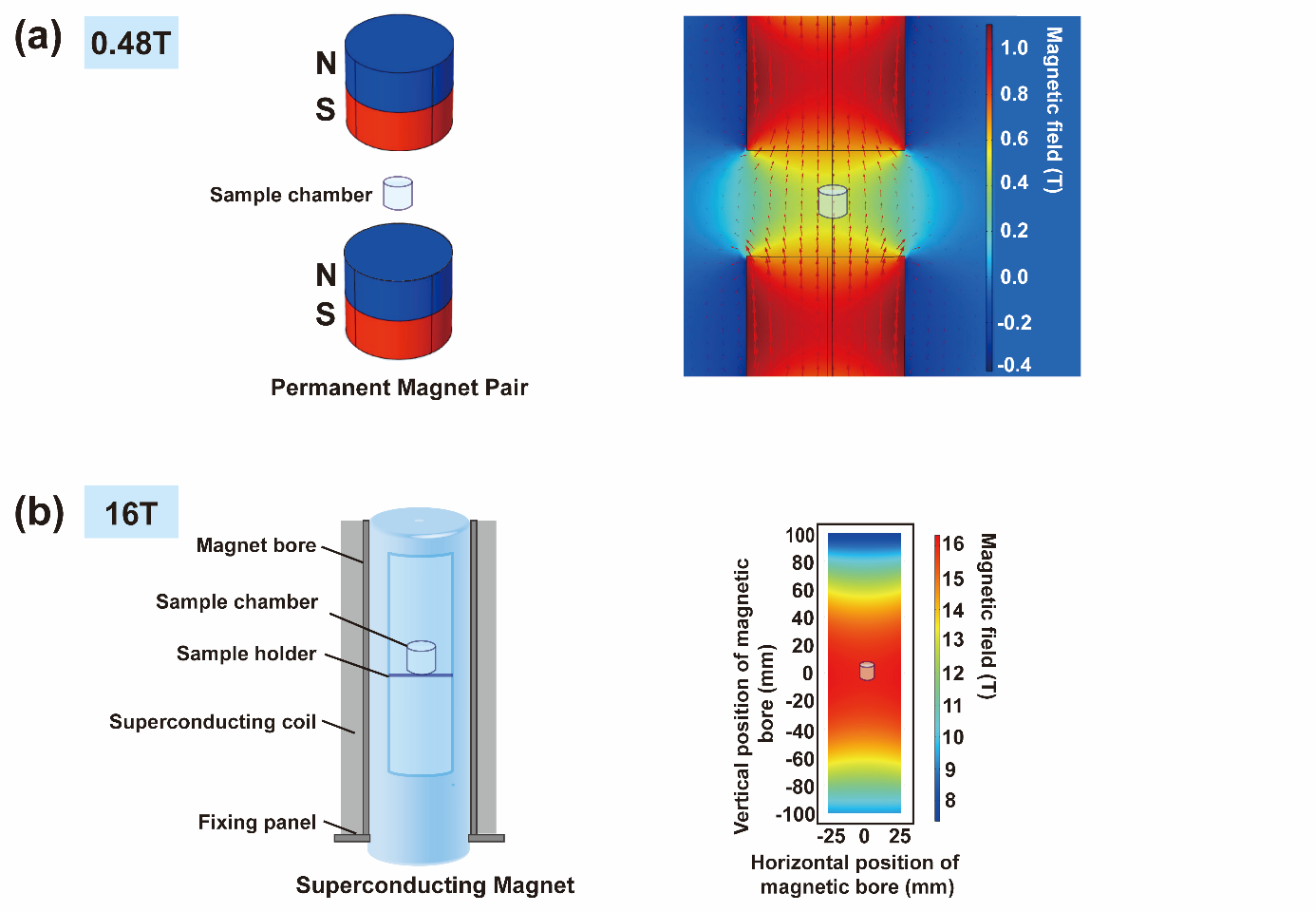


**Figure S4. Schematic illustration and magnetic field distribution of the magnets.** (a) Permanent magnet pair. (b) Superconducting magnet.


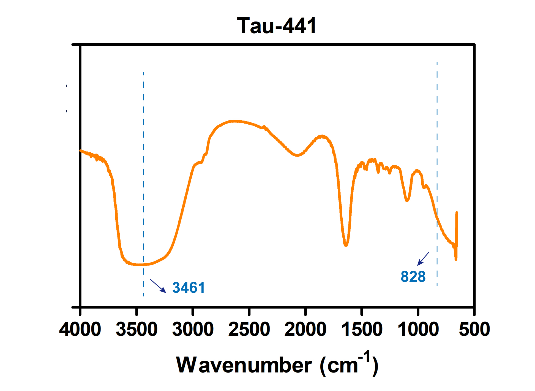


**Figure S5. Fourier transform infrared spectroscopy (FTIR) spectra of native Tau-441 solution at 310.15 K.**


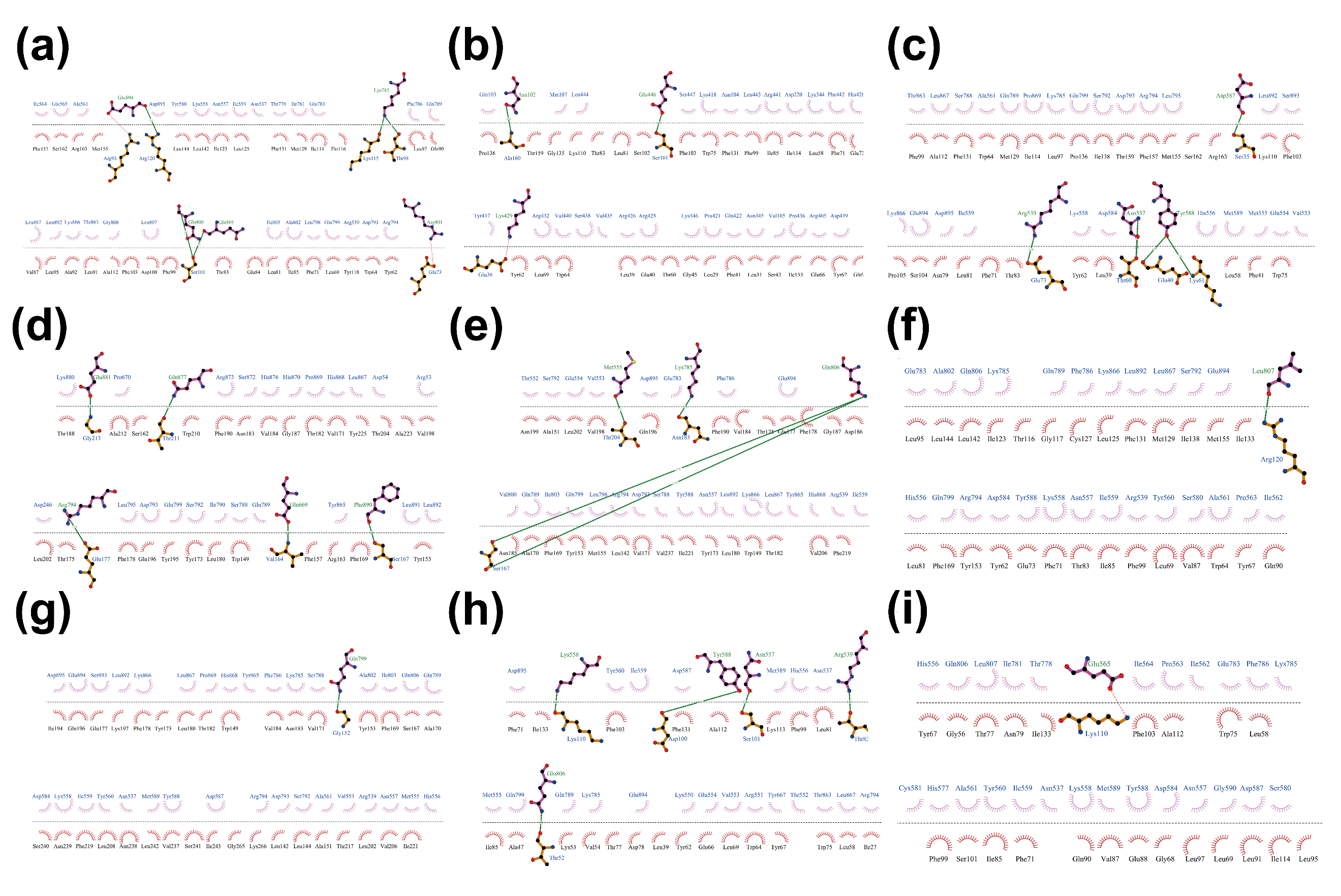


**Figure S6.** **Putative binding sites between voltage-dependent anion channel (VDAC I) and hexokinase (HK).** The top nine possible binding sites of HK binding to VDAC I shown by ZDOCK and Ligplot software. The dotted green line indicates hydrogen bonds, and the arc with spokes radiating toward the ligand atoms indicates hydrophobic interactions.


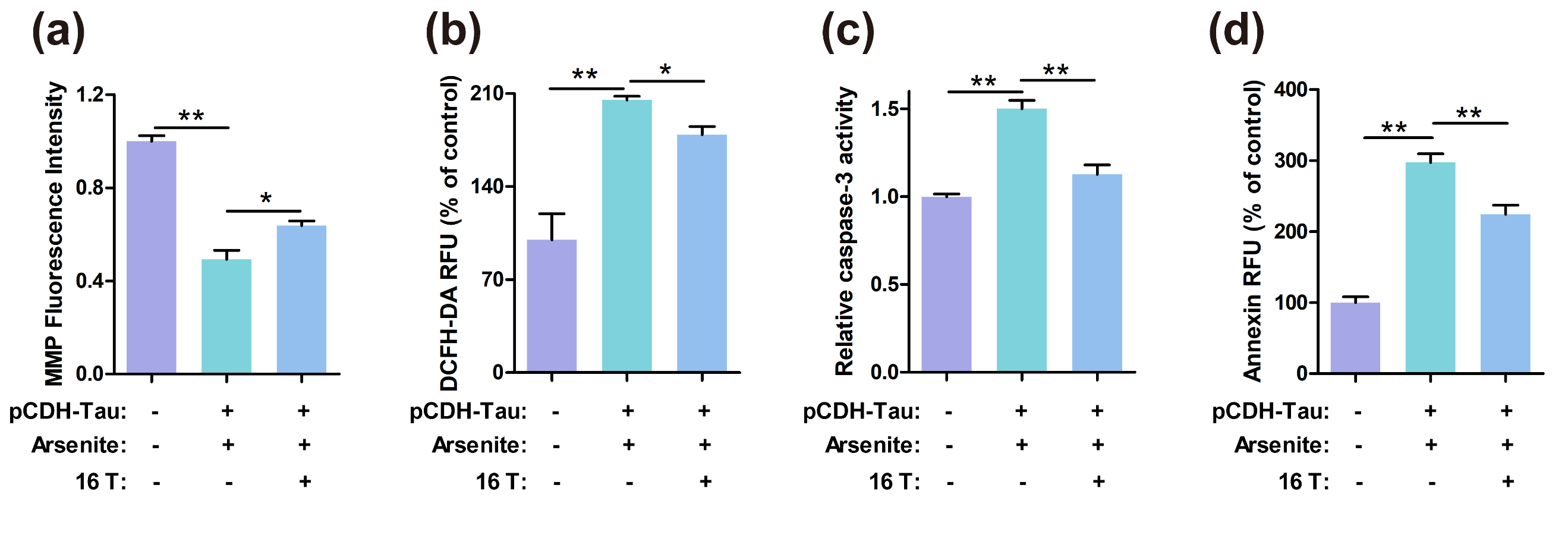


**Figure S7. Apoptosis of 293T**-**TAU441 cells was decreased by an MF (16 T) induction for 6 h.** (a) Mitochondrial membrane potential (MMP) of 293T-TAU441 with and without an MF (16 T) measured by a fluorescence spectrophotometer. (b) DCFH-DA fluorescence intensity representing ROS generation in 293T-TAU441 cells with and without an MF (16 T). (c) Caspase-3 activity in 293T-TAU441 cells with and without an MF (16 T) measured by a fluorescence of Ac-DEVD-pNA. (d) Cell apoptosis with and without an MF (16 T) detected by Annexin V-PE. Data were shown as mean ± SEM. *n* = 3 per group. * *p* < 0.05, ** *p* < 0.01.


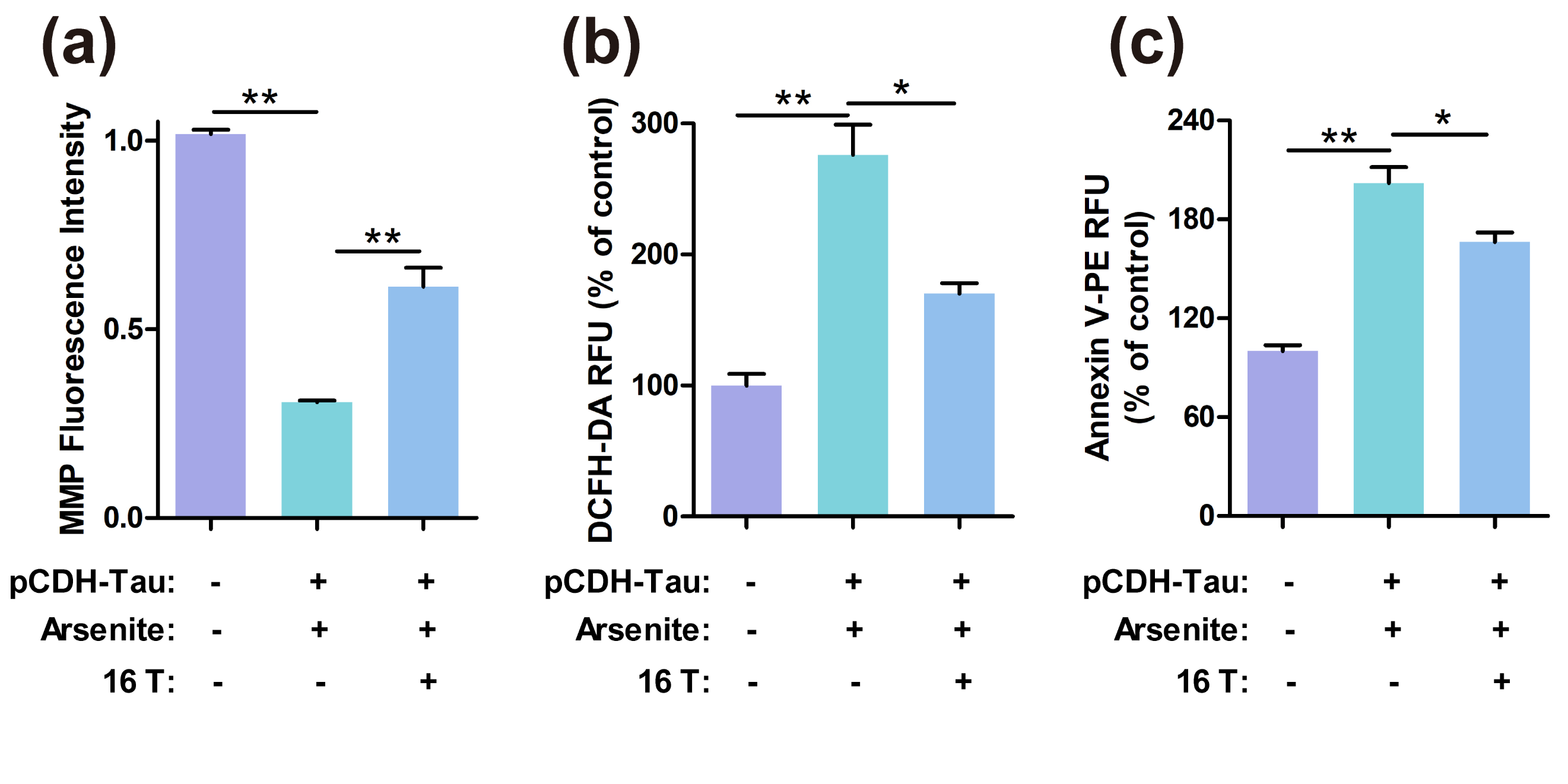


**Figure S8. Apoptosis of SK-N-SH-TAU441 cells was decreased by an MF (16 T) induction for 6 h.** (a) Mitochondrial membrane potential (MMP) of SK-N-SH-TAU441 cells with and without an MF (16 T) measured by a fluorescence spectrophotometer. (b) DCFH-DA fluorescence intensity representing ROS generation in SK-N-SH-TAU441 cells with and without an MF (16 T). (c) Cell apoptosis with and without an MF (16 T) detected by Annexin V-PE. Data were shown as mean ± SEM. *n* = 3 per group. * *p* < 0.05, ** *p* < 0.01.


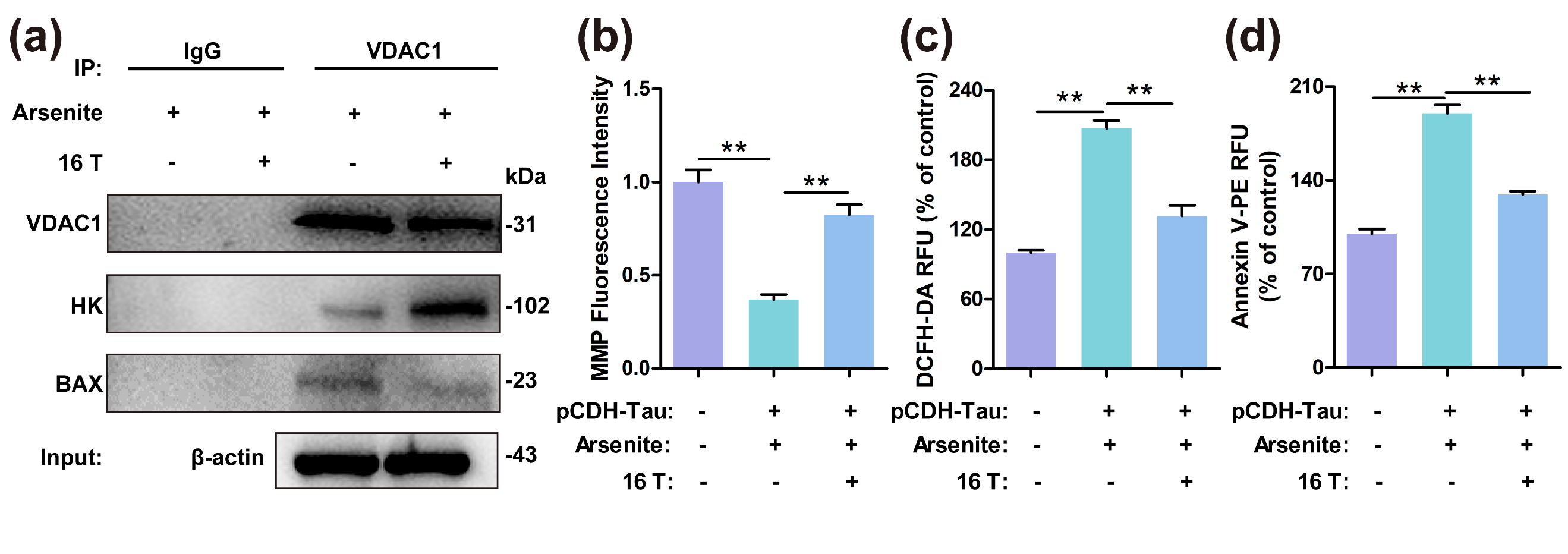


**Figure S9. Apoptosis of** **SK-N-SH-TAU441 cells was decreased by an MF (16 T) induction for 24 h.** (a) Western blotting of VDAC I, HK, Bax, and β-actin expression in lysate and Co-IP of SK-N-SH-TAU441 cells. (b) Mitochondrial membrane potential (MMP) of SK-N-SH-TAU441 cells with and without an MF (16 T) measured by a fluorescence spectrophotometer. (c) DCFH-DA fluorescence intensity representing ROS generation in SK-N-SH-TAU441 cells with and without an MF (16 T). (d) Cell apoptosis with and without an MF (16 T) detected by Annexin V-PE. Data were shown as mean ± SEM. *n* = 3 per group. * *p* < 0.05, ** *p* < 0.01.**Supporting Tables**

**Table S1.** Top 10 possible solvation free energy gains upon hexokinase binding to native Tau-441 calculated by the molecular docking method

| Number | ΔG (kcal/mol) |
| --- | --- |
| 1 | -14.3 |
| 2 | -7.7 |
| 3 | -3.7 |
| 4 | -1.4 |
| 5 | -20.6 |
| 6 | -4.3 |
| 7 | -4.5 |
| 8 | -6.7 |
| 9 | -7.6 |
| 10 | -5.2 |

**Table S2.** Top 10 possible solvation free energy gains upon hexokinase binding to voltage-dependent anion channel calculated by the molecular docking method

| Number | ΔG (kcal/mol) |
| --- | --- |
| 1 | -14 |
| 2 | -23.6 |
| 3 | -9 |
| 4 | -19.4 |
| 5 | -17 |
| 6 | -21.1 |
| 7 | -21.8 |
| 8 | -18.5 |
| 9 | -16.3 |
| 10 | -15.6 |

**SI References**

[1] Rankin CA, Sun Q, Gamblin TCJMBR. (2005) Pseudo-phosphorylation of tau at Ser202 and Thr205 affects tau filament formation. *Molecular Brain Research*. 138 (1), 84–93.
